# Lysosomal Re-acidification Prevents Lysosphingolipid-Induced Lysosomal Impairment and Cellular Toxicity

**DOI:** 10.1371/journal.pbio.1002583

**Published:** 2016-12-15

**Authors:** Christopher J. Folts, Nicole Scott-Hewitt, Christoph Pröschel, Margot Mayer-Pröschel, Mark Noble

**Affiliations:** Department of Biomedical Genetics, University of Rochester School of Medicine and Dentistry, Rochester, New York, United States of America; Baylor College of Medicine, UNITED STATES

## Abstract

Neurodegenerative lysosomal storage disorders (LSDs) are severe and untreatable, and mechanisms underlying cellular dysfunction are poorly understood. We found that toxic lipids relevant to three different LSDs disrupt multiple lysosomal and other cellular functions. Unbiased drug discovery revealed several structurally distinct protective compounds, approved for other uses, that prevent lysosomal and cellular toxicities of these lipids. Toxic lipids and protective agents show unexpected convergence on control of lysosomal pH and re-acidification as a critical component of toxicity and protection. In *twitcher* mice (a model of Krabbe disease [KD]), a central nervous system (CNS)-penetrant protective agent rescued myelin and oligodendrocyte (OL) progenitors, improved motor behavior, and extended lifespan. Our studies reveal shared principles relevant to several LSDs, in which diverse cellular and biochemical disruptions appear to be secondary to disruption of lysosomal pH regulation by specific lipids. These studies also provide novel protective strategies that confer therapeutic benefits in a mouse model of a severe LSD.

## Abstract 2

Different structurally related lipids from three genetically distinct lysosomal storage disorders are sufficient to cause multiple dysfunctions that converge on disruption of lysosomal pH as a core pathogenic mechanism.

## Author Summary

Neurodegenerative lysosomal storage disorders (LSDs) are severe and untreatable recessive genetic disorders that cause devastating damage to the nervous system. These diseases exhibit severe disruption of lysosomes (a cellular organelle that breaks down lipids and proteins) and other aspects of cell function. However, the means by which mutations cause these dysfunctions are poorly understood. By studying different lipids that accumulate in three different LSDs, we found that lipids with specific shared structures are sufficient to cause multiple lysosomal and cellular dysfunctions, including an abnormal alkalization of the lysosomal pH. We prevented all of these dysfunctions by promoting lysosomal re-acidification and discovered several drugs—already approved for other purposes—with unexpected abilities to restore lysosomal pH and rescue cells. In a genetic mouse model of a severe LSD, one of these compounds decreased tissue damage, improved quality of life, and extended survival. In contrast with previous studies on individual disorders, our study provides novel shared principles relevant to several LSDs and uncovers relevant compounds able to provide multiple benefits in a disease-relevant model in vivo.

## Introduction

Lysosomal storage disorders (LSDs) represent some of the most difficult of medical challenges, with poorly understood pathologies and only rare treatment options. Despite having the common property of being caused by mutations in lysosomal enzymes, leading to accumulation of substances that would normally be degraded and to more generally compromised lysosomal function, the more than 40 different LSDs differ greatly in their primary tissue pathology, their severity, and in the specific substances that accumulate within compromised cells. The individuality of these diseases is mirrored by the dominant therapeutic strategies, which are generally focused on replacement of missing enzyme activity (by protein administration or gene expression) or on substrate reduction therapies that have the goal of decreasing availability of a precursor for the substance whose degradation is compromised by enzyme mutation [1–39]. Such therapies have proven useful in rare cases [40–43], but progress on therapeutic advances is infrequent and essentially nonexistent for LSDs exhibiting damage to the central nervous system (CNS) [44–46]. In addition, progress has tended to be disease specific rather than providing principles that may apply more broadly.

Despite extensive study of LSDs, many critical questions remain unanswered about these diseases. For example, little is known about the biochemical linkage between any particular mutation and lysosomal dysfunction, or even whether there is a direct correlation between accumulation of particular substances and lysosomal dysfunction. In addition, although both lysosomal dysfunction and cellular dysfunctions occur in these diseases, it remains unclear how—or even if—these changes are functionally connected. Moreover, it is unclear whether principles that might be relevant to an individual disease are relevant to the pathology of diseases caused by different mutations.

To attempt to discover principles that might be relevant to LSDs caused by different mutations, we have focused on diseases associated with accumulation of lipids that are able to cause a variety of cellular dysfunctions, up to and including cell death, when applied to cells in vitro. Such diseases include Krabbe disease (KD), metachromatic leukodystrophy (MLD), and Gaucher disease [22, 31, 47–55]. Although each of these diseases is associated with accumulation of a different lipid (or lipids) and with different disease pathologies, the effects of these lipids on cellular function are severe enough to suggest that such toxicities may contribute to disease pathogenesis.

We now show that a structurally related subset of lipids that accumulate in KD, MLD, or Gaucher disease all induce multiple lysosomal dysfunctions (along with other cellular dysfunctions), thus providing a direct link between enzymatic mutations and lysosomal abnormalities. We further show that it is possible to use drug-repurposing assays to discover single compounds that block a wide range of lipid-induced toxicities. Analysis of the properties of toxic lipids and of protective compounds reveals a previously unsuspected role of lysosomal pH and re-acidification as a potentially valuable therapeutic target. We further provide proof of principle that selecting potential therapies based on their ability to improve lysosomal function without correcting a genetic defect can reveal compounds that offer clinically relevant benefits in a mouse model of a severe LSD.

## Results

### Psychosine Disrupts Multiple Cellular Functions in Oligodendrocyte Progenitor Cells

We began our studies with an analysis of psychosine (Psy, also referred to as galactosylsphingosine), a lipid that is thought to be of central, and potentially causal, pathogenic importance in KD [56–60]. Psy is one of the most extensively studied of all the lipids known to accumulate in LSDs and is known to exhibit toxicity for multiple cell types in vitro [61–75] and to cause extensive damage when injected intracranially in wild-type (WT) mice [59]. Psy accumulates in tissues of individuals with KD due to galactocerebrosidase ([GALC], EC 3.2.1.46) mutations that cause abnormal processing of lipids that are important components of myelin, the insulating material that enwraps axons in the CNS and peripheral nervous systems (PNS), a primary target of damage in KD. Psy also accumulates in tissues of the naturally occurring, severe murine model of KD, the *twitcher* mouse [76–80], which also harbors GALC mutations and recapitulates most human pathologies.

**Fig 1:**
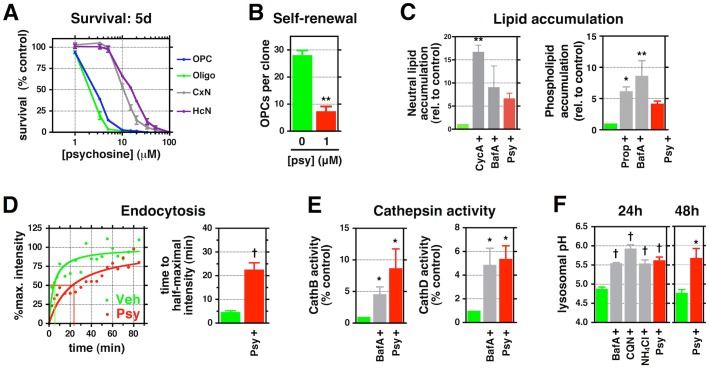
Psy causes a diverse array of cellular and biochemical toxicities in cultured O-2A/OPCs. **(A)** Survival of Psy-treated purified rat O-2A/OPCs, OL, cortical, and hippocampal neurons for 5 d relative to untreated controls. **(B)** Quantification of the number of rat O-2A/OPCs per clone in the presence or absence of Psy over 5 d. **(C)** Quantification of the relative amount of neutral lipids and phospholipids in rat O-2A/OPCs exposed to positive controls cyclosporin A (10 μM) or propranalol (10 μM), 100 nM bafilomycin A (BafA), or 3.3 μM Psy for 48 h. **(D)** Quantification of time to half-maximal intensity for rat O-2A/OPCs exposed to Psy (1 μM) or vehicle (DMSO) for 24 h before addition of fluorescent nanobeads. A plot of relative intensity is also shown; lines indicate time to half-maximal intensity. **(E)** Quantification of relative Cathepsin B and D activities in rat O-2A/OPCs exposed to the indicated drugs or 1 μM Psy for 24 h. **(F)** Quantification of lysosomal pH in rat O-2A/OPCs exposed to 500 nM BafA, 10 μM chloroquine (CQN), 10 mM ammonium chloride (NH_4_Cl), or 1 μM Psy for 24 h or 48 h. Data for all graphs displayed as mean ± standard error of the mean (SEM); *p < 0.05, **p < 0.01, ^†^*p* < 0.001 versus control. Data presented in this figure can be found in S1 Data.

As a prelude to analyzing the ability of Psy to alter cellular function, we first determined which CNS cells were most vulnerable to this lipid and found that the most sensitive cells were primary oligodendrocyte (OL)/type-2 astrocyte progenitor cells ([O-2A/OPCs], also referred to as OL precursor cells). These progenitors, which give rise to the myelin-forming OLs of the CNS during development and in response to myelin damage, were killed by pathophysiologically relevant low-micromolar (3 μM) concentrations of Psy [77] that had no effect on hippocampal and cortical neuron survival (see also, e.g., [65, 81]) and were as toxic to OLs (Fig 1A). The vulnerability of primary O-2A/OPCs to Psy was also an order of magnitude greater than seen in immortalized CNS glial progenitor cell lines (e.g., [82]) and in Schwann cells of the PNS [83]. This level of sensitivity falls well within the reported Psy concentrations in the CNS of symptomatic (postnatal day [P]25) and moribund (P40) *twitcher* mice, which are between 15 μM and 34 μM, respectively [77]. Similar concentrations in the *twitcher* CNS have been reported by other investigators, ranging from 0.7 μM (P10), 4 μM (P16), and 4.5 μM (P20–P25) to as high as 27–50 μM (P30) [78–80]. Comparable concentrations have been reported in postmortem Krabbe patient CNS tissue, ranging from 2.7 μM to 45 μM in cortical grey and white matter, respectively [84, 85].

Further studies revealed that O-2A/OPCs exposed to still lower (1 μM) levels of Psy exhibited multiple abnormalities of potential relevance to understanding the decreased myelination and apparent failure to repair myelin damage seen in KD. In the absence of cell death, 1 μM Psy suppressed both cell division (Fig 1B) and differentiation of O-2A/OPCs into OLs (S1A Fig). It also disrupted cytoskeletal integrity and caused decreased cell migration (S1B and S1C Fig). Such sensitivity places these cells among those most sensitive to the effects of Psy exposure.

### Psy Alters Lysosomal Function in O-2A/OPCs

We next discovered that exposure to 1 μM Psy has the previously unrecognized ability to cause multiple alterations in lysosomal function, indicating that this lipid may provide a direct link between enzymatic mutation and lysosomal dysfunction in KD.

Exposure to Psy caused abnormalities in lipid homeostasis, endolysosomal transport, and cathepsin activity. Exposure to 1μM Psy disrupted lipid homeostasis, causing the intracellular accumulation of both neutral triglycerides and phospholipids (Fig 1C, S1D Fig). Endolysosomal transport was also compromised by exposure to 1 μM Psy, as shown by a decreased rate of endocytic import of fluorescently labeled polystyrene nanobeads (time to half-maximal staining intensity: 4.6 ± 1.0 min for vehicle-treated control versus 22.2 ± 5.7 min for Psy, *p* < 0.05; Fig 1D, S1E Fig). Psy exposure also increased the activity of resident lysosomal proteases cathepsin D and B, which can cause cellular damage or death upon export to the cytoplasm and the activities of which are known to be elevated in a number of LSDs (Fig 1E) [86–88]. Psy’s ability to disrupt lysosomal function was as great as that seen with bafilomycin A (BafA), which disrupts lysosomal function by antagonizing the lysosomal vacuolar-type H^+^-ATPase [89].

Exposure of O-2A/OPCs to 1 μM Psy significantly increased intralysosomal pH from 4.88 ± 0.04 to 5.62 ± 0.08 after 24 h, an increase maintained for at least 48 h after a single exposure (*p* < 0.001; Fig 1F). This elevation in lysosomal pH was observed in both fixed (Fig 1F) and live (S1F Fig) O-2A/OPCs. Psy exposure was as potent at increasing lysosomal pH as multiple compounds well known to exert such effects, including BafA, chloroquine, or the weak base NH_4_Cl (Fig 1F) [90]. This increase was evident within minutes of exposure to Psy and was comparable to treatment with BafA (S1G Fig, S1–S3 Movies), and the effects on lysosomal pH were sustained over 24–48 h after Psy exposure.

### Unbiased Screening Identifies Chemically Diverse Candidate Protective Agents That Prevent Psy-Induced Cellular and Lysosomal Dysfunctions

**Fig 2:**
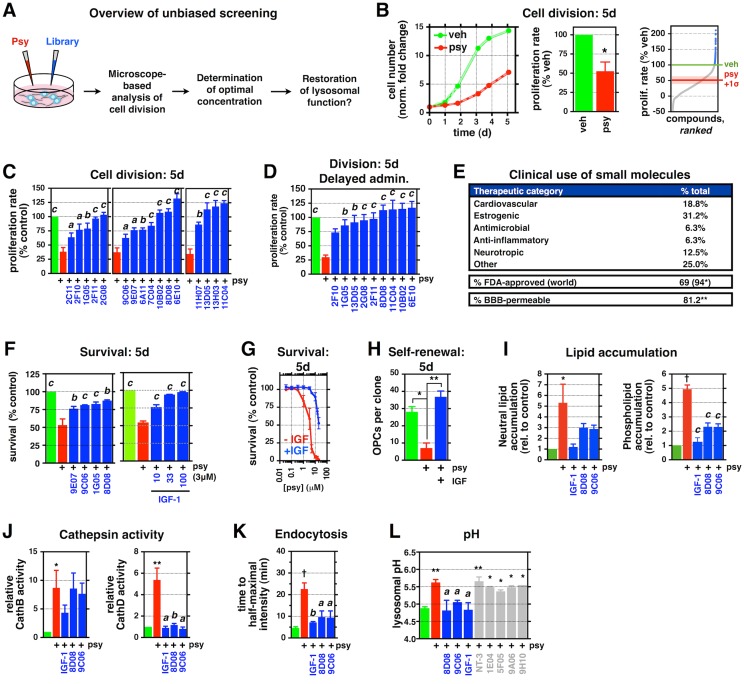
Unbiased screening identifies chemically diverse candidate protective agents that reduce Psy toxicities. **(A)** Schematic depicting the work flow for unbiased screening with Celigo adherent cytometer (Nexcelom). **(B)** Representative plot of relative cell number over 5 d for rat O-2A/OPCs exposed to 1 μM Psy or vehicle (DMSO). Quantification of the relative proliferation rate for all 1,040 drugs at three concentrations over 5 d. Blue: “hit” drugs selected for further validation. A bar graph quantifying the relative proliferation rate of all vehicle and Psy controls is included. **(C)** Quantification of the relative proliferation rate of rat O-2A/OPCs exposed to 1.5 μM Psy, with and without verified pro-division “hits,” over 5 d. **(D)** Quantification as in **(C)** for cells exposed to Psy 48 h before exposure to the indicated drugs. **(E)** Summary of clinical metadata for lead “hits” as % of total. (* worldwide approval, including FDA. ** reported blood–brain barrier permeability; not all drugs have been examined/reported.) **(F)** Quantification of cell survival in cells exposed to 3.3 μM Psy for 5 d, with and without the indicated drugs, for 5 d. **(G, H)** Quantification of the **(G)** relative survival and **(H)** number of rat O-2A/OPCs per clone exposed to of Psy (1 μM in **H**), with and without 100 ng/mL insulin-like growth factor (IGF-1), for 5 d. **(I–K)** Quantification of **(I)** neutral lipid and phospholipid accumulation, **(J)** cathepsin B and D activities in rat O-2A/OPCs exposed to the indicated drugs, with and without 1 μM Psy, and **(K)** time to half-maximal intensity for endocytosis of fluorescent nanobeads for **(I)** 2 d or **(J, K)** 24 h. **(L)** Quantification of lysosomal pH for rat O-2A/OPCs exposed to 1 μM Psy, with and without the indicated “hits” (blue) or “non-hits” (gray), for 24 h. NT-3: 10 ng/mL; 1E04: 5 μM; 5F05, 9A06, 9H10: 1 μM. ^a^*p* < 0.05, ^b^*p* < 0.01 versus Psy-only. Data for all graphs displayed as mean ± SEM; *ns* = not significant; *p < 0.05, **p < 0.01, ^†^*p* < 0.001 versus untreated; ^a^*p* < 0.05, ^b^*p* < 0.01, ^c^*p* < 0.001 versus Psy-only treatment. See also S2 Fig, S1 and S2 Tables for drug names and concentrations. Data presented in this figure can be found in S1 Data.

To identify potential means of preventing Psy-induced toxicities that might be suitable for eventual clinical utilization, we conducted unbiased analysis of multiple concentrations of 1,040 mostly United States Food and Drug Administration (FDA)-approved small molecules [91] and a custom panel of 12 growth factors with known neuroprotective activity. We examined prevention of Psy-induced suppression of O-2A/OPC division in these analyses (Fig 2A).

We found 16 structurally and functionally diverse compounds (S2A Fig, S1 and S2 Tables), in addition to 4 growth factors, that had the unexpected properties of rescuing cell division (Fig 2B and 2C, S2B Fig). Eight of the 9 most protective agents were effective at rescuing cell division even when their administration was delayed 48 h after Psy exposure (Fig 2D). Importantly, all small molecules were optimally protective at physiologically achievable concentrations (i.e., nanomolar to low micromolar), and most are approved for use in humans (94%) and are blood–brain barrier permeable (>80%) (Fig 2E).

Five agents (chlorotrianisene [1G05], NKH-477 [(9C06), also known as colforsin], clofoctol [8D08], tulobuterol [9E07], and insulin-like growth factor [IGF-1]) revealed by our screens not only significantly rescued cell division but were also able to reduce cell death caused by exposure to higher concentrations of Psy (Fig 2F). With the exception of IGF-I, none of our compounds of interest were previously identified as being able to protect against toxicity of Psy (or of other lipids accumulating in LSDs). Even in the case of IGF-I, previous studies reported that supraphysiological (>10 μg/mL) concentrations decreased Psy-induced apoptosis in OLs [92] and in an O-2A/OPC cell line [82]. In our studies, by contrast, 100 ng/mL IGF-1 shifted Psy’s cytotoxicity curve by an order of magnitude and significantly deceased Psy-dependent suppression of self-renewal (Fig 2G and 2H).

Further examination of three of the most protective agents—clofoctol, NKH-477, and IGF-I—demonstrated that these compounds also prevented Psy-induced alterations in lysosomal function. All three suppressed Psy-induced increases in lipid accumulation and cathepsin activity and restored normal endocytosis (Fig 2I–2K). Moreover, all three compounds significantly decreased Psy-dependent increases in pH (Fig 2L). In contrast, five randomly selected molecules that did not significantly reduce any Psy toxicities in our screens (caffeine [1E04], acetarsol [5F05], mepartricin [9A06], avobenzone [9H10], and neurotrophin-3; S1 Table) had no effect on Psy-induced increases in lysosomal pH.

### Protective Agents Converge on a Limited Number of Common Necessary Pathways for Their Activity

**Fig 3:**
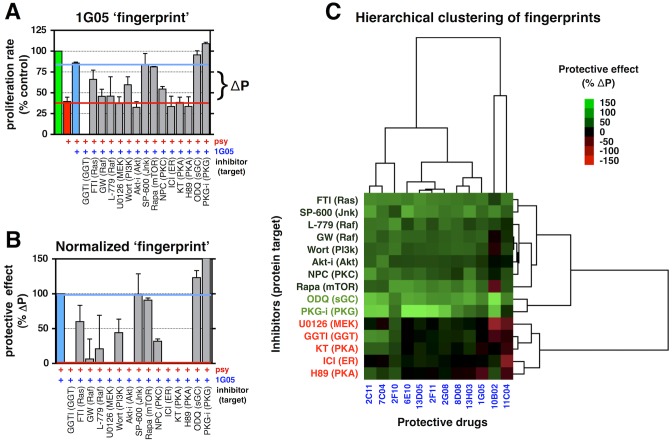
Protective agents converge on a limited number of common necessary pathways for their activity. **(A, B)** Representative “fingerprint of protection” for candidate drug 1G05. Quantification of the relative proliferation rate of rat O-2A/OPCs exposed to 1.5 μM Psy, with and without 100 nM 1G05, and with and without pharmacological inhibitors targeting the indicated protein, for 5 d. To account for differences in their ability to reduce Psy-induced suppression of division between candidate agents, relative changes in cell division were normalized as in **(B)**. **(C)** Unsupervised hierarchical clustering of “fingerprints” for the indicated drugs. Data for all graphs displayed as mean ± SEM. See also S1 and S2 Tables for drug names and concentrations, and S3 Table for details on the “fingerprinting” screen. Data presented in this figure can be found in S1 Data.

As the protective compounds we discovered are structurally and functionally diverse, we next attempted to define regulatory pathways on which these agents might converge to confer their protective activity. To do this, we focused on multiple signaling pathways previously described to be antagonized by exposure of cells to Psy (e.g., mitogen-activated protein kinase [MAPK], phosphoinositide-3-kinase [PI3k]/Akt, protein kinase C [PKC], cyclic Amp (cAMP)-dependent signaling [51, 74, 82, 93–95]), as well as other proteins that have been implicated in mediating stress responses in O-2A/OPCs, including Jun N-terminal kinase (Jnk) [74], mammalian target of rapamycin (mTOR) [96–98], and estrogen receptor [99–101]. We next generated a secondary screen consisting of pharmacological inhibitors to components of these various signaling pathways. In these experiments, O-2A/OPCs were exposed to Psy; a combination of Psy and a protective agent; or a combination of Psy, a protective agent, and one of 15 pharmacological inhibitors targeting important signaling pathways and proteins (S3 Table). This allowed for the generation of a compound-specific “fingerprint of protection” that revealed putative signaling pathways used by the candidate compound to overcome Psy-induced suppression of division (e.g., Fig 3A and 3B).

We generated fingerprints for 12 of the most efficacious compounds in reducing Psy-induced suppression of division across 5 d using this approach. The results were then hierarchically clustered to identify similarities and dissimilarities between individual compound fingerprints and between the signaling pathways implicated in protection (Fig 3C). Despite structural and functional diversity among candidate protective agents, there was striking similarity in the signaling pathways needed for protection.

The activity of the diverse protective agents was antagonized by pharmacological inhibition of the Ras/rapidly accelerated fibrosarcoma gene (Raf)/MAPK pathway, Akt, estrogen receptor, protein kinase A (PKA), and geranylgeranyl transferase ([GGT], which is needed for activation of small GTPases that are involved in cell division and migration). Despite their structural diversity, there was a surprisingly high degree of correlation between groups of small molecules; the cluster of structurally and functionally unrelated drugs 2G08 (ethopropazine, an antiparkinsonian drug), 2F11 (estradiol valerate, a synthetic estrogen), and 8D08 (clofoctol, an antibiotic), for example, showed the highest degree of similarity (correl. = 0.97) (S3 Fig).

### Protection Against Psy-Induced Lysosomal and Cellular Dysfunctions Can Be Provided by Promotion of Lysosomal Re-acidification

Lysosomal ion homeostasis, maintained through the activity of several channels and transporters, is critical to the normal function of lysosomes. For example, H^+^ import is necessary for the maintenance of an acidic pH [102] and is achieved through the activity of the V-ATPase, Ca^2+^ is important for vesicle trafficking [103] and fusion [104], Na^+^ and K^+^ are required for the regulation of membrane potential [105, 106], and Cl^−^ serves as a counterion to regulate lysosomal membrane potential and to facilitate the acidification of the lysosome lumen [107–109]. Although any of these may be potential therapeutic targets, we focused our attention on identifying those channels or transporters regulated by signaling pathways uncovered through our fingerprinting analysis.

**Fig 4:**
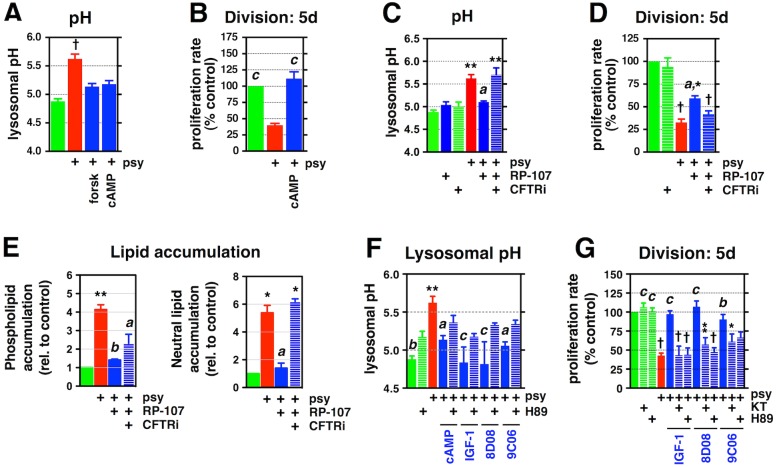
Promotion of lysosomal re-acidification is critical in protecting from multiple aspects of Psy toxicity. **(A–D)** Quantification of the lysosomal pH and proliferation rate in rat O-2A/OPCs exposed to **(A, C)** 1 μM Psy for 24 h, **(B, D)** 1.5 μM for 5 d, with and without **(A, B)** 1 mM cAMP (or 10 μM forskolin), or **(C, D)** 333 nM RP-107 and 1 μM cystic fibrosis transmembrane conductance regulator (CFTR) inhibitor 172 (CFTRi-172). **(E)** Quantification of neutral lipid and phospholipid accumulation in rat O-2A/OPCs exposed to the indicated drugs, with and without 1 μM Psy, for 2 d. **(F, G)** Quantification of the lysosomal pH and proliferation rate in rat O-2A/OPCs exposed to 1 μM Psy for **(F)** 24 h or **(G)** 5 d, with and without 100 nM KT-5720 or 3.3 μM H89 (cAMP: 1 mM). Data for all graphs displayed as mean ± SEM; *p < 0.05, **p < 0.01, ^†^*p* < 0.001 versus untreated; ^a^*p* < 0.05, ^b^*p* < 0.01, ^c^*p* < 0.001 versus Psy-only treatment. See also S1 and S2 Tables for drug names and concentrations. Data presented in this figure can be found in S1 Data.

Of the several pathways that are required for activity of our protective agents, the one for which there is a clearly established linkage to at least one aspect of lysosomal function is the requirement for PKA activity. Previously, it has been shown that cAMP can promote lysosomal re-acidification [110], as can PKA, which is activated by cAMP [111]. In addition, we found that increases in cAMP not only normalized lysosomal pH but also prevented Psy-induced decreases in O-2A/OPC division (Fig 4A and 4B), raising the theoretical possibility that intervention at this point would provide additional benefits beyond that of pH restoration. We therefore focused attention on the role of lysosomal re-acidification as a potential therapeutic target.

The most attractive explanation for how cAMP/PKA activity could restore lysosomal pH would be through activation of the cystic fibrosis transmembrane conductance regulator (CFTR), a PKA-activated transmembrane chloride channel that promotes lysosomal re-acidification [112]. Unlike the CLC-7 Cl^−^/H^+^ antiporter, another chloride channel that is localized to the lysosomal membrane and thought to play a role in the basal maintenance of lysosomal pH [113], the CFTR channel appears only to be critical for re-acidification. Moreover, although the CFTR can be activated by PKA and cAMP, there is no evidence for such activation of CLC-7. In addition, specific agonists and inhibitors exist for the CFTR, enabling a direct test of whether promoting re-acidification can prevent Psy-induced toxicity.

As we predicted, treatment of cells with the cAMP-independent CFTR agonist RP-107 [114] restored lysosomal pH in cells exposed to Psy. Although control of lysosomal pH and/or lysosomal re-acidification has not been thought to have any upstream role in the multiple cellular dysfunctions caused by Psy exposure, we nonetheless found that RP-107 protected against Psy-induced suppression of division, as well as elevated storage of both neutral lipids and phospholipids (Fig 4C–4E). To test the hypothesis that these benefits were not due to off-target effects of RP-107, we also co-exposed cells to CFTR-inhibitor 172 (CFTRi-172) [115], which attenuated the protective effects of RP-107 treatment (Fig 4C–4E).

The effects of RP-107 were CFTR dependent, as determined by knockdown of *CFTR* in O-2A/OPCs using small interfering RNA (siRNA) pools targeting rat *CFTR*, as well as a pool of nontargeting (NT) siRNAs as a control for transfection; the reduction in CFTR protein levels was confirmed by western blot analysis. Knockdown of *CFTR* did not significantly affect lysosomal pH when compared to cells exposed to NT controls (4.96 ± 0.13 versus 4.81 ± 0.11, respectively). Moreover, in the presence of Psy, in both NT and *CFTR* siRNA pools, there was a significant increase in lysosomal pH (5.47 ± 0.08 versus 5.61 ± 0.09, respectively), with no significant difference between these two treatment groups. However, when we tested the effect of RP-107, a specific CFTR agonist, we found that lysosomal pH was significantly reduced in cells exposed to NT siRNA but that *CFTR* knockdown attenuated RP-107’s protective effect (5.18 ± 0.09 versus 5.62 ± 0.03, *p* < 0.01; S4A Fig). Thus, as with our pharmacological experiments, genetic loss of CFTR does not appear to significantly affect basal lysosomal pH in untreated cells. However, the protective capacity of RP-107 is CFTR dependent. These results are consistent with the original studies demonstrating the role of the CFTR in control of lysosomal re-acidification [112].

Moreover, we found that the most effective protective agents identified in our studies did not themselves reduce the basal acidic pH of lysosomes in the absence of Psy (S4B Fig) but instead seemed to work to promote re-acidification. Indeed, their ability to normalize lysosomal pH, as well as rescue cell division, in cells exposed to Psy was blocked by co-exposure to inhibitors of PKA (Fig 4F and 4G). As these protective agents are able to rescue cells even when applied 48 h after Psy exposure (Fig 2D), it appears that their protective activity is not mediated simply by blocking lysosomal alkalization.

### Psy’s Free Amine Group Is Critical for Its Toxicity

The observations that multiple Psy-induced lysosomal and cellular dysfunctions can be prevented by lysosomal re-acidification with RP-107 (Fig 4C–4E) and that Psy exposure causes rapid increases in lysosomal pH (Fig 1F, S1D Fig), raise complementary questions about how Psy causes such changes. One possibility is that Psy disrupts the function of particular proteins involved in lysosomal re-acidification, but another possibility is that structural features of Psy itself are directly relevant to understanding effects on lysosomal pH.

Although multiple studies have attempted to understand the molecular mechanisms underlying Psy’s toxicity [61–75], we noted that Psy has unusual physicochemical features that might be of relevance to understanding its effects on lysosomes. Psy is unusual as a cationic, weakly basic lipid, carrying a net positive charge at physiological pH. With a pKa value of 7.18 [116], Psy is predicted to be 99.9% protonated in the acidic pH of the lysosome. If this aspect of Psy’s structure is important in altering lysosomal and cellular function, then the protonatable free amine group on Psy should be critical in mediating the changes in lysosomal pH that we observed. We therefore tested whether removing this free amine group altered effects on lysosomal pH and on other outcomes of Psy exposure.

**Fig 5:**
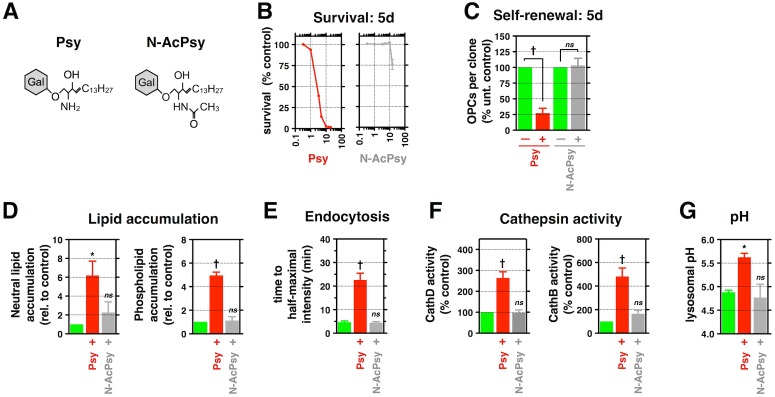
Psy’s free amine group is critical for its toxicity. **(A)** Structures of Psy and N-AcPsy. **(B)** Quantification of rat O-2A/OPC survival in response to Psy or N-AcPsy. **(C–G)** Quantification of rat O-2A/OPC self-renewal, lipid accumulation, endocytosis, cathepsin activity, and lysosomal pH in cells exposed to 1 μM Psy or N-AcPsy for **(C)** 5 d, **(D)** 2 d, or **(E–G)** 24 h. Data for all graphs displayed as mean ± SEM; *ns* = not significant; *p < 0.05, ^†^*p* < 0.001 versus control. Data presented in this figure can be found in S1 Data.

We found that the free amine group on Psy is critical in its ability not only to disrupt lysosomal pH but also to cause other toxic effects. We compared Psy toxicity to that of N-acetyl-Psy (N-AcPsy), a structural derivative containing an amide-linked acetyl group, rendering it no longer protonatable (Fig 5A). Unlike Psy, N-AcPsy did not induce cell death or alter O-2A/OPC self-renewal at similar concentrations (Fig 5B and 5C). Moreover, N-AcPsy did not elevate neutral lipid and phospholipid storage, increase endocytic transport time, increase cathepsin activity, or elevate lysosomal pH (Fig 5D–5G). Thus, the positively charged free amine group present on Psy was critical to increasing lysosomal pH and also to the subsequent lysosomal and cellular impairments observed after exposure in O-2A/OPCs.

### Structurally Related Lysosphingolipids from Multiple LSDs Alter Lysosomal Function

**Fig 6:**
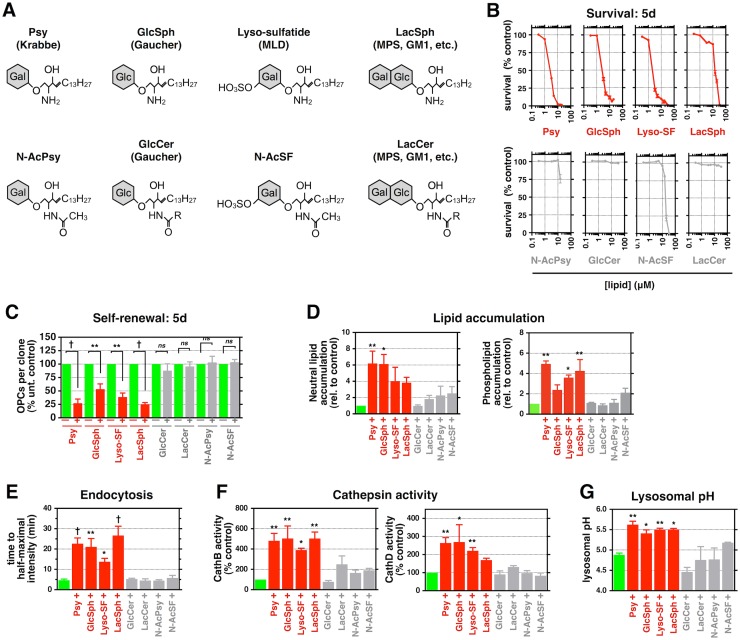
Lysosphingolipids accumulating in other LSDs suppress critical O-2A/OPC behaviors and lysosomal function. **(A)** Chemical structures of the indicate lipids. Gal: galactose; Glc: glucose; R: variable hydrocarbon chain. **(B–G)** Quantification of **(B)** the relative survival, **(C)** the relative number of O-2A/OPCs per clone, **(D)** lipid accumulation, **(E)** endocytic import time, **(F)** cathepsin activity, and **(G)** lysosomal pH in rat O-2A/OPCs exposed to the indicated lipids for **(B and C)** 5 d, **(D, E, G)** 24 h, or **(F)** 2 d. Data for all graphs displayed as mean ± SEM; *ns* = not significant; *p < 0.05, **p < 0.01, ^†^*p* < 0.001 versus untreated, unless otherwise indicated. S4 Table for lipid concentrations used in **(C–G)**. Data presented in this figure can be found in S1 Data.

To further test the hypothesis that the presence of free amine group on a cationic lipid is critical to lipid-induced toxicities, and that such lipids provide a direct link between enzymatic mutation and lysosomal disruption, we examined a series of lipids known to accumulate in other LSDs. Other lipids of potential interest include lyso-sulfatide (lyso-SF) (which accumulates in MLD [49]), glucosylsphingosine (GlcSph) and glucosylceramide (GlcCer) (which accumulate in Gaucher disease [47]), and lactosylsphingosine (LacSph) and lactosylceramide (LacCer), which accumulate in several LSDs (Fig 6A) [78, 117, 118]. Some of these lipids appear to have been only rarely studied for their effects on cell function in vitro (lyso-SF, GluSph, LacSph, LacCer) [119]. In the case of Gaucher disease, the majority of previous in vitro studies appears to have focused on GlcCer, and studies on both GlcCer and GlcSph often have required lipid concentrations severalfold greater than those at which Psy’s effects were observed (e.g., [22, 50, 51, 53, 55, 120–122]).

In order to eliminate differences in cell types as potential contributors to different outcomes, we examined the survival and self-renewal of O-2A/OPCs exposed to lyso-SF, GlcSph, GlcCer, LacSph, and LacCer. Use of these cells also provided a test of the hypothesis that the structure of a lipid is of primary importance in determining toxicity. We also examined the effects of N-acetyl-sulfatide (N-AcSF) as a direct comparison with N-AcPsy. We found that sphingosine-derived lipids that accumulate in different LSDs and that contain a free amine group (and thus are structurally similar to Psy) caused significant cell death and suppression of self-renewal (Fig 6B and 6C) at similarly low lipid concentrations as we observed with Psy. In contrast, exposure to their ceramide-based counterparts GlcCer and LacCer, or to N-AcSF, did not cause cellular toxicities at comparable or 10-fold higher concentrations (Fig 6B and 6C).

We also found that lysosphingolipids accumulating in other LSDs [22, 31, 47–55, 123–127] had similar effects as Psy on lysosomal function. Exposure to sublethal concentrations of GluSph, lyso-SF, or LacSph caused increases in neutral lipid and phospholipid accumulation, endocytic transport time, cathepsin activity, and lysosomal pH. In contrast, exposure to their non-lyso counterparts did not have such effects (Fig 6D–6G).

If the hypotheses are correct that other toxic lysosphingolipids that accumulate in LSDs work through similar mechanisms as Psy, and that such mechanisms are relevant to understanding the efficacy of our protective agents, then our protective agents also should rescue cells from the toxic effects of lipids from other LSDs. If correct, such findings would provide both the first structural predictors of toxicity and the first example of protective agents of potential relevance in different LSDs.

**Fig 7:**
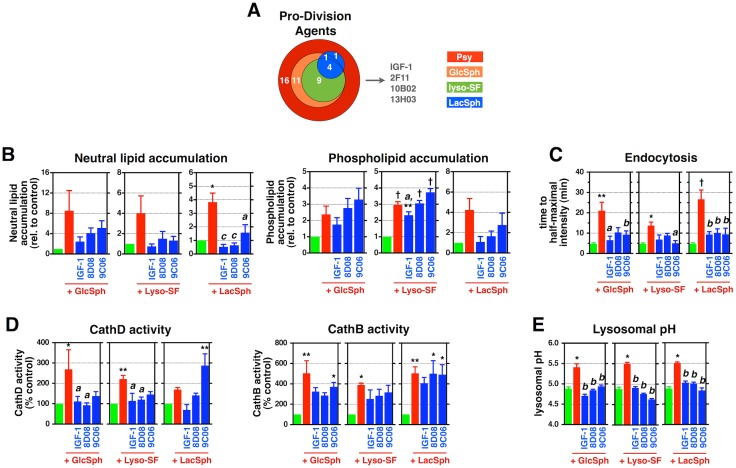
Candidate protective agents reduce multiple lysosphingolipid-induced lysosomal and cellular toxicities. **(A)** Venn diagram summarizing the number of protective drugs, including IGF-1, that reduce suppression of division in rat O-2A/OPCs exposed to the indicated lyso-lipids for 5 d. **(B–E)** Quantification of **(B)** lipid accumulation**, (C)** endocytic import time, **(D)** cathepsin activity, and **(E)** lysosomal pH in rat O-2A/OPCs exposed to the indicated lyso-lipids, with and without 100 ng/mL IGF-1, 100 nM 8D08, or 333 nM 9C06, for **(B)** 2 d or **(C–E)** 24 h. Data for all graphs displayed as mean ± SEM; *ns* = not significant; *p < 0.05, **p < 0.01, ^†^*p* < 0.001 versus untreated; ^a^*p* < 0.05, ^b^*p* < 0.01 versus Psy-only treatment, unless otherwise indicated. See also S4 Fig and S1 and S2 Tables for drug names and concentrations and S4 Table for lipid concentrations. Data presented in this figure can be found in S1 Data.

We found that our candidate protective agents also reduced the toxic effects of GlcSph, lyso-SF, and LacSph (Fig 7A). Three of our most effective agents—IGF-1, clofoctol (8D08), and NKH-477 (9C06)—prevented lipid-induced suppression of division and also attenuated increases in neutral lipid and phospholipid accumulation, endocytic import time, cathepsin activities, and lysosomal pH in rat O-2A/OPCs exposed to sublethal concentrations of GlcSph, lyso-SF, or LacSph (Fig 7B–7G, S5A Fig). These agents also rescued cell division in cells exposed to GlcSph or Lyso-SF for 48 h before addition of protective agents (S5B Fig).

### Lysosphingolipids Disrupt Human OL Progenitor Cell Behaviors, and Protective Compounds Rescue Human Cells

We next examined the question of whether the principles revealed in our studies on cells derived from the CNS were applicable to human cells. In these experiments, we used an anti-CD140a (PDGFRα) antibody to enrich for a population of human O-2A/OPCs from the corpus callosal field of mid-gestation fetal tissue (S6 Fig) [128] and exposed cells to Psy and potential protective agents as for rat-derived cells.

**Fig 8:**
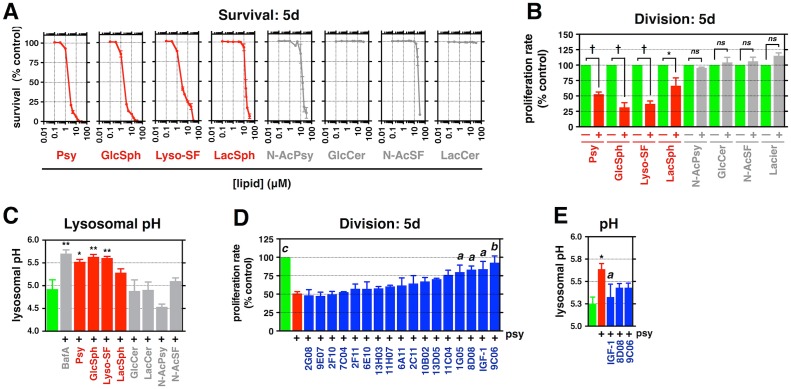
Lysosphingolipids disrupt human O-2A/OPC behaviors. **(A–C)** Quantification of **(A)** the relative survival, **(B)** the relative proliferation rate, and **(C)** lysosomal pH of human O-2A/OPCs exposed to the indicated lipids for **(A, B)** 5 d or **(C)** 24 h. BafA: 100 nM. **(D, E)** Quantification of the relative proliferation rate and lysosomal pH in human O-2A/OPCs exposed to 1 μM Psy for **(D)** 5 d or **(E)** for 24 h, with and without the indicated drugs. ^a^*p* < 0.05 versus Psy-only. Data for all graphs displayed as mean ± SEM; *ns* = not significant; *p < 0.05, **p < 0.01, ^†^*p* < 0.001 versus untreated control; ^a^*p* < 0.05, ^b^*p* < 0.01 versus Psy-only treatment. See also S5 Fig and S1 and S2 Tables for drug names and concentrations, and S4 Table for lipid concentrations. Data presented in this figure can be found in S1 Data.

Exposure to lysosphingolipids caused death in human cells at concentrations comparable to those used in rat progenitor cells (S4 Table), as well as suppression of cell division and elevation of lysosomal pH at sublethal concentrations, whereas their non-lyso counterparts did not cause similar toxicities (Fig 8A–8C). Notably, cell division and normalization of lysosomal pH were restored in cells exposed to Psy with clofoctol, NKH-477, and IGF-I, as we observed for rat O-2A/OPCs (Fig 8D and 8E).

### Alterations in O-2A/OPC Biology in Twitcher Mice Are Like Those Induced By Psy Exposure In Vitro

In the final section of our studies, we asked whether discoveries made on WT cells exposed exogenously to Psy in vitro revealed principles applicable to cells with an LSD-relevant mutation, both in respect to cellular pathologies and to rescue of lysosomal function. These studies were carried out using *twitcher* mice, a naturally occurring model of KD that recapitulates most human symptoms. Multiple studies have demonstrated that this mouse is a reliable model of KD in respect to enzymatic dysfunction and tissue pathology [77–80, 129, 130] and is also one of the most useful models for studying LSDs in general. In particular, *twitcher* mice progress from a lack of apparent pathology to severe disease over a relatively rapid time course, with function appearing to be normal at birth, followed by disease symptoms manifesting about 20 d after birth and with death ensuing at about 42 d after birth. This time course allows pathology and the effects of treatment to be studied at different stages of disease progression.

**Fig 9:**
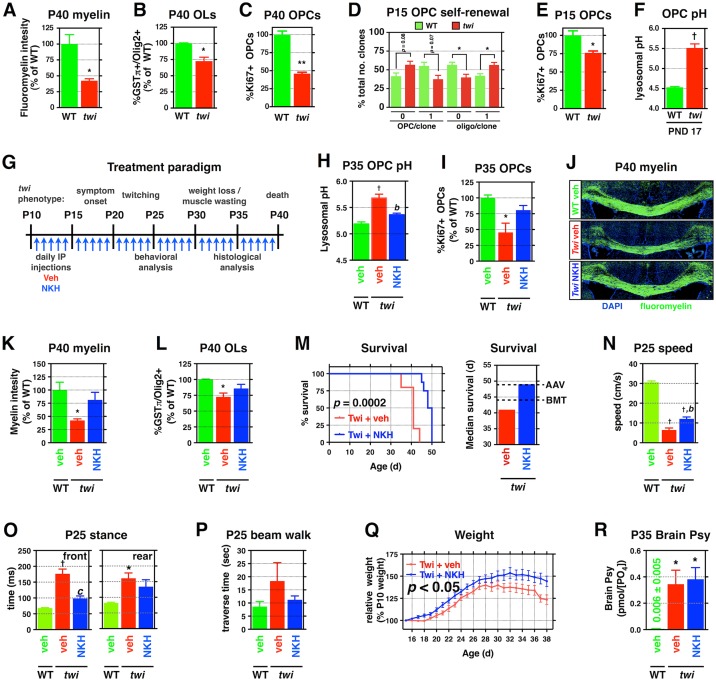
NKH-477, a protective compound identified in vitro, protects against multiple toxicities in treated *twitcher* mice. (**A–C)** Quantification of **(A)** fluoromyelin intensity, **(B)** number of GSTpi+/Olig2+ OLs, and **(C)** the relative number of dividing Ki67+/Olig2+ O-2A/OPCs in the corpus callosa of P40 *twitcher* mice and age-matched WT littermates (*n* = 3 from different litters). **(D)** Analysis of clonal composition of P15 *twi* O-2A/OPCs and WT littermates across 5d. **(E)** Quantification of the relative number of dividing Ki67+/Olig2+ O-2A/OPCs in the corpus callosa of P15 *twitcher* mice and age-matched WT littermates (*n* = 3 from different litters). **(F)** Quantification of lysosomal pH of O-2A/OPCs acutely isolated from P17 *twitcher* and WT mice. **(G)** Overview of treatment paradigm and clinical course for *twitcher* mice. **(H)** Quantification of lysosomal pH of O-2A/OPCs isolated from P35 treated mice. **(I)** Quantification of the number of dividing callosal O-2A/OPCs in P35 treated mice. **(J–L)** Representative confocal images of fluoromyelin-stained corpus callosa of the indicated treatment groups at P40, in addition to quantification of staining intensity and the number of OLs. **(M)** Kaplan–Meyer survival curve for treated and untreated *twi* mice. Median survival of *twi* mice, with dotted lines indicating reported median survival of single-therapy treatments [15]. **(N–Q)** Quantification of travel speed, stance time, beam traverse time, and relative weights for P25 saline-treated WT and *twi*, as well as NKH-477–treated *twi*, mice. **(R)** Quantification of brain Psy levels at P35. Data for all graphs displayed as mean ± SEM; *ns* = not significant; *p < 0.05, ^†^*p* < 0.001 versus WT; ^b^*p* < 0.01 versus vehicle-treated *twi*. Data presented in this figure can be found in S1 Data.

In our studies on *twitcher* mice, we first determined that changes in O-2A/OPC function were like those induced by exposure to low doses of Psy in vitro. We found significant reductions in both myelin content and OL cell number (OLs; Olig2^+^/GST^+^) in the corpus callosum—the major myelinated tract of the CNS—at P40 when compared to age-matched WT littermates (Fig 9A and 9B), consistent with previous analyses of human and *twitcher* tissue [76, 131]. We also observed a significant reduction in the percentage of dividing (Ki67^+^) O-2A/OPCs (54.0% ± 1.9% of WT, *p* < 0.01; Fig 9C) at this late time point, during which time O-2A/OPCs should be undergoing rapid expansion through cell division to replace damaged OLs and myelin.

We additionally found that O-2A/OPC function was compromised in presymptomatic *twitcher* mice in ways similar to those induced by Psy exposure. We isolated O-2A/OPCs from presymptomatic P15 *twitcher* mice and examined their self-renewal capacity in vitro. These cells showed impaired self-renewal in comparison with cells of age-matched WT cells when maintained in vitro for 5 d (Fig 9D). Such findings were mirrored by significant reductions in the pool of dividing O-2A/OPCs in vivo at P15 (Fig 9E).

To determine whether cells harboring a mutant GALC gene exhibit changes in lysosomal pH, we examined the endolysosomal pH of corpus callosal O-2A/OPCs acutely isolated from presymptomatic *twitcher* mice (P17). We found that the lysosomal pH was significantly less acidic than that of cells isolated from age-matched WT littermates (Fig 9F), similar to what was observed in vitro with exogenous Psy treatment (Fig 1F). Thus, O-2A/OPCs isolated at developmental time points in which symptoms are not obvious (prior to P18–20) show altered lysosomal pH and alterations in critical cellular behaviors like those induced by exposing WT cells to Psy in vitro.

### NKH-477 Protects against Multiple Toxicities in Twitcher Mice

We next investigated whether the analytical approach employed in our in vitro studies could identify compounds able to provide clinically relevant benefits in vivo. We focused our studies on NKH-477 (9CO6), a water-soluble derivative of forskolin that is approved for treatment of acute heart failure in Japan [132], as this agent is known to be CNS penetrant and elevates cAMP levels (through direct activation of adenylyl cyclase) in brains of rats after systemic administration [133]. Moreover, unlike the other identified protective agents, the linkage of NKH-477 to PKA regulation (and thus to lysosomal re-acidification) is both defined and mediated through widely expressed proteins, consequentially not requiring cells to express specialized drug-targeted receptors in order to be responsive.

We initiated treatment at P10, a time when CNS concentrations of Psy are already approaching the range at which we see effects on O-2A/OPCs [78–80], using once-daily intraperitoneal (IP) injections (1 mg/kg; Fig 9G). This is a point in time when disruptions in neuronal function can already be observed in *twitcher* mice [134], raising the possibility of initiating treatment only after subtle clinical changes are first observable. This delayed initiation of treatment is in marked contrast with the well-studied need to initiate the application of bone marrow transplantation and/or gene therapy in the first few days after birth in order to obtain benefit [1, 10, 16, 40, 130].

The primary endpoints of interest in our in vivo studies were whether we could rescue lysosomal and cellular function in O-2A/OPCs and whether once-daily treatment with NKH-477 is sufficient to provide benefit on both parameters. O-2A/OPCs were isolated at P35 to examine the effects of NKH-477 treatment on lysosomal pH, and we found a normalization of pH in cells isolated from treated *twitcher* mice when compared to vehicle-treated mice (Fig 9H). NKH-477–treated *twitcher* mice also showed an increase in the numbers of dividing O-2A/OPCs at P35 to near-normal levels, as well as increases in myelin content and increased OL cell numbers at moribund ages, when compared to vehicle-treated *twitcher* mice, again to levels not significantly different from WT mice (Fig 9I–9L).

Remarkably, we also found that NKH-477 treatment provided significant lifespan extension that was comparable to published single-therapy treatments aimed at restoring GALC activity, including bone marrow transplantation (the current standard of care in patients) or viral-mediated gene therapy (Fig 9M) [1, 2, 10, 13, 15, 16]. Moreover, *twitcher* mice that received daily injections of NKH-477 also showed significantly improved locomotor and gait function (Fig 9N–9P, S7 Fig) and significantly improved weight gain throughout their lifespan when compared to vehicle-treated *twitcher* littermates (Fig 9Q). These benefits were observed despite the fact that we were not correcting the genetic defect; indeed, we did not find that NKH-477 treatment reduced the overall tissue burden of Psy in the CNS (Fig 9R).

## Discussion

Our studies provide multiple novel findings related to the biology of LSDs. We found in both rodent and human cells that structurally related sphingolipids that accumulate in these disorders appear to directly cause multiple lysosomal dysfunctions. We also discovered multiple pharmacological agents, previously approved for other clinical purposes, that prevent all of the sphingolipid-induced lysosomal and cellular toxicities we analyzed, apparently by promoting lysosomal re-acidification. In vivo studies in the *twitcher* mouse model of KD demonstrated the ability of one of the agents we identified, which is known to be CNS penetrant, to correct lysosomal pH in O-2A/OPCs, as well as to provide multiple therapeutically relevant benefits in the absence of correcting the underlying genetic mutations implicated in the disease.

The finding that pathophysiologically relevant low levels of four different sphingolipids known to accumulate in different LSDs are each sufficient to compromise multiple lysosomal functions appears to provide the first evidence that substances created due to mutations of lysosomal enzymes may be directly responsible for initiating the metabolic dysfunctions that characterize such diseases. Previous studies have speculated that lysosomal dysfunction is caused by such events as intralysosomal accumulation of substances that are not properly degraded (e.g., [19, 20, 24, 26, 28, 30, 32, 135–137]), but we could find no prior demonstration—or suggestion—that a specific substance known to accumulate in LSDs is able to simultaneously alter lysosomal pH, endolysosomal trafficking, lipid degradation, and cathepsin activation.

Based on the comparative structures of toxic and nontoxic lipids, we hypothesize that toxicity is caused by disruption of lysosomal pH. All of the four toxic lipids we studied share the presence of a theoretically protonatable free amine group, raising the possibility that their accumulation increases the net positive charge in the lysosomal lumen, altering ion homeostasis and decreasing acidification by suppressing proton influx through the V-type H^+^ ATPase. In contrast, such effects were not caused by other lipids that accumulate in these disorders and that lack this free amine group (i.e., GlcCer, LacCer), or by lysosphingolipids with an acetylated amine group (N-AcPsy and N-AcSF) attenuated toxicity.

The only remotely comparable studies we could find to our own were those of Sillence and colleagues [120, 121], who reported that exposure of the virally transformed tumorigenic RAW murine macrophage cell line to 40 μM GlcCer for 48 h altered trafficking of boron-dipyrromethene (BODIPY)-labeled LacCer to the lysosome, and that an unspecified concentration of GlcCer caused modest increases in lysosomal pH in these cells. However, such studies also demonstrated that exposure to 20 μM GlcCer or GlcSph decreased lysosomal pH in RAW cells exposed to a GlcCer synthase inhibitor, that such effects were not caused by exposure to Psy, and that these GlcCer and GlcSph concentrations caused negligible cell death [120, 121]. Thus, these previous results differ markedly from those obtained in our studies examining effects of 10- to 20-fold lower concentrations of Psy, GlcSph, lyso-SF, and LacSph and also do not indicate that lipids with similar structures cause similar lysosomal or cellular pathologies. In addition, although studies on sphingosine (applied at 10-μM concentrations) suggested the free amine group on this lipid is important for its toxicity, these studies considered the role of the amine group was to confer detergent-like properties on sphingosine and did not consider potential relevance to control of lysosomal pH [138, 139].

The possibility that changes in lysosomal pH may be of particular importance in understanding the effects of exposure to toxic sphingolipids, and that lysosomal neutralization may be upstream of multiple lysosomal and cellular dysfunctions and may provide a novel therapeutic target, was strongly supported by our findings that we rescued O-2A/OPCs from adverse effects of lipid exposure by activation of the CFTR (which promotes lysosomal re-acidification [112]). Exposure to RP-107, a chemical activator of CFTR [114], prevented alterations in lysosomal pH and also rescued cells from adverse effects on division in a CFTR-dependent manner. In addition to CFTR, there are likely several other lysosomal targets that may be relevant for treatment. Accumulation of undegraded sphingomyelin, for example, has been shown to alter membrane trafficking and lysosomal calcium homeostasis through the impairment of the TRPML1 channel [140].

We think it is also important not to interpret our findings as indicating that activation of chloride flux via the CFTR will be the sole mechanism available for promoting lysosomal re-acidification, or that regulation of chloride flux is the only possible way to promote restoration of a normally acidic pH. The CFTR provides a well-studied protein for which there is strong data indicating a role in re-acidification [112], for which useful experimental drugs are available, and for which a role of PKA in activation has been identified. But it seems likely there will be other proteins that offer potential entry points for promoting re-acidification. In respect to the much more studied problem of the control of basal lysosomal pH, there is strong disagreement on whether chloride ion flux through the CFTR or through CLC chloride channels or Cl^−^/H^+^ exchangers is central to controlling basal lysosomal acidification. Expression of mutant CFTR in alveolar macrophages was reported to be associated with a lack of proper acidification of their degradative compartments [141, 142]. In contrast, other authors found that the CFTR was not required for phagolysosomal acidification in macrophages [143] or respiratory epithelial cells [144], with other investigators also questioning the importance of CFTR in promoting lysosomal acidification [145]. These disagreements also extend to the CLC chloride channels or Cl^−^/H^+^ exchangers, and some investigators have reported that loss of CLC-7 is not associated with alterations in lysosomal acidification [107, 108, 146]. Moreover, there are also intriguing observations that cation transport may also be important in the regulation of basal lysosomal pH [102, 143]. Although it may be that some of these disagreements arise due to use of different techniques [113], it may also be the case that there are nuances of lysosomal regulation that differ in different cell types, and also that lysosomal re-acidification may be regulated by flux of cations or anions other than chloride. The extent to which controversies regarding control of basal lysosomal pH are pertinent to studies on control of lysosomal re-acidification is not yet known, however. We hope that the results of our present studies will further increase interest in this important problem and will lead to identification of other regulatory pathways of potential therapeutic relevance.

Although some of the effects of individual toxic sphingolipids that we studied have been observed previously with other cell types (although usually at higher lipid concentrations than we utilized, e.g., [51, 61–75, 93, 95, 119–122, 147–154]), there is no previous indication that all of these forms of damage may ultimately be controlled by a single metabolic parameter or that such a parameter might control lysosomal pH. It is also worth noting that, although multiple mechanisms have been observed to contribute to particular effects of Psy or of other toxic lipids [51, 61–75, 82, 120, 123, 147–150, 152–187], none has demonstrated the ability to correct the multiple dysfunctions prevented by promotion of lysosomal re-acidification.

Additional support for the hypothesis that control of lysosomal pH is of central importance in understanding the pathology of toxic lipid exposure was provided by the identification, by unbiased drug screening, of novel protective agents that show no known prior overlap in function but that all converged on promoting lysosomal re-acidification. We found that clofoctol, NKH-477, and IGF-1 all restored lysosomal pH in lipid-exposed cells, despite having no known common properties. Restoration of lysosomal pH appears to be due to promotion of re-acidification, as none of these protective agents acidified lysosomes in the absence of Psy. As the only known convergence of the protective substances we identified (including RP-107) is a common ability to promote lysosomal re-acidification, it currently is most likely that it is this aspect of their effects that is most important.

**Fig 10:**
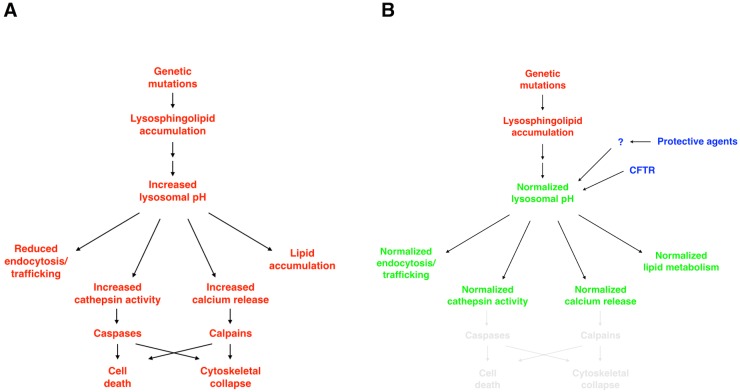
Hierarchical targeting of lysosomal pH by candidate protective compounds reduces multiple lysosphingolipid-induced cellular dysfunctions. We propose a model in which genetic mutations in resident lysosomal enzymes result in the accumulation of lysosphingolipids, which increase endolysosomal pH and consequently disrupt lysosome-dependent cellular processes, including endocytosis, ion homeostasis, lipid metabolism, and enzymatic activities. Candidate protective drugs—as well as direct activation of CFTR—converge on their abilities to normalize lysosomal pH, apparently through re-acidification processes, and reduce disruptions of downstream cellular processes, including survival and cellular division.

The possibility that regulation of lysosomal pH and re-acidification could represent a convergence point of disease pathology and therapeutic intervention for LSDs appears to be novel. Interest is emerging in the possibility that promoting lysosomal re-acidification may offer therapeutic benefit in situations of lysosomal dysfunction, but studies thus far have been focused only on the possibility that restoring normal lysosomal pH will enhance normal protein degradation [32, 110, 111, 188–192]. Nonetheless, the possibility that regulation of lysosomal pH could represent a central mechanism in disease pathogenesis and treatment is consistent with the dependence of normal lysosomal function on an acidic pH (as summarized in Fig 10). For example, neutralization can cause release of lysosomal Ca^2+^ and cathepsins [32, 192]: Ca^2+^ release could compromise cytoskeletal function [193] and hence cell division, whereas cathepsin release and activation could initiate cell death [194]. In addition, increasing lysosomal pH would be predicted to decrease function of any lysosomal enzymes evolutionarily optimized for function in an acidic environment.

The question of whether it was possible to modify lysosomal and cellular function in an animal model of a severe LSD was studied by administering NKH-477 to *twitcher* mice, a severe murine model of an LSD that exhibits a pattern of disease progression similar to that seen in KD patients [195] and that is the most widely used model for studying possible disease interventions [1–17]. NKH-477 was the logical choice for such studies, as it was the one small molecule protective agent identified thus far with known CNS penetrance [133] and with a well-defined drug target. In contrast with studies on gene therapy and/or bone marrow transplantation, both of which would be constant in their effects and generally must be initiated shortly after birth when animals are presymptomatic to obtain benefit in experimental models [1, 2, 10, 13, 15, 16], we only administered NKH-477 once daily beginning 10 d after birth. This starting point was chosen both because this is a time when CNS concentrations of Psy first begin to approach those utilized in our in vitro studies and to test the hypothesis that our approaches could identify interventions able to provide benefit even when initiated after it might be possible to detect early changes in neuronal function [65–67].

The primary goal of our in vivo studies was to determine whether NKH-477 administration could be used to normalize lysosomal pH and improve O-2A/OPC function. After first confirming that O-2A/OPCs isolated from twitcher mice showed similar abnormalities as WT progenitor cells treated with Psy in vitro, we found that daily treatment with NKH-477 normalized lysosomal pH (as analyzed ex vivo in O-2A/OPCs isolated from treated and control twitcher mice) and rescued O-2A/OPC division in vivo.

Benefits of daily NKH-477 treatment extended far beyond rescue of O-2A/OPC division and lysosomal pH and offered several clinically relevant improvements. At the cellular level, daily NKH-477 administration rescued OL numbers and myelin content even at a time when vehicle-treated littermates were moribund. Moreover, mice treated with NKH-477 showed improved motor behavior and weight gain (suggesting that cell types other than O-2A/OPCs also benefitted from this treatment). In addition, survival was significantly extended, even to the same degree previously reported with gene therapy alone (and exceeding that obtained with bone marrow transplants alone) [1, 2, 10, 13, 15, 16]. These multiple benefits were obtained even though we did not correct the underlying genetic defect nor decrease the overall Psy tissue burden, and there was no prior information on using this compound (or any related compounds) in the context of LSDs. Although NKH-477 increases cAMP levels in the CNS [133] and could thus have other effects beyond promotion of lysosomal re-acidification, the fact that NKH-477 shares the property of promoting re-acidification with the other compounds we identified, and was indeed able to rescue lysosomal pH and O-2A/OPC division in vivo, makes it seem likely that this aspect of drug action is at least partially relevant to the benefits observed.

Even if some of the in vivo benefits we observed were due to other activities of NKH-477 than promotion of lysosomal re-acidification, this would not decrease interest in this agent as a potential candidate for further analysis. Recent reviews of the outcomes of implementing newborn screening (NBS) for detection of early infantile KD (EIKD) in New York state [196] have led to the conclusion that, “in addition to the potential harm to families receiving false-positive test results, NBS for EIKD appears to have resulted in a reduction in survival in individuals who have the disease. The data from the New York program suggest that NBS for EIKD should be abandoned, pending the development of improved screening or therapies shown to confer both survival and quality-of-life benefits over supportive care. The results of this experience suggest that research efforts should be focused on improving presymptomatic treatment outcomes in children identified by NBS prior to the redeployment of mandatory presymptomatic screening" [197].

As treatment with NKH-477 confers both survival and quality-of-life benefits in the established animal model for KD and already has been approved (in Japan) for use in humans [132], this may provide an attractive starting point for thinking about new approaches to some of the devastating LSDs with severe neuropathology. Moreover, the discovery of mechanisms and protective strategies that apply to distinct lipids accumulating in three different LSDs provides hope that these same general principles will apply to other LSDs characterized by lysosphingolipid accumulation, and perhaps also in other LSDs (such as the neuronal ceroid lipofuscinoses/Batten disease) in which lysosomal pH is abnormally more alkaline [198]. In addition, the ability of re-acidification to rescue a diverse range of lysosomal and cellular dysfunctions raises the question of whether similar strategies might provide broadly useful effects in important diseases in which lysosomal dysfunction has also been implicated, such as Alzheimer’s and Parkinson’s disease (e.g., [199–212]).

## Materials and Methods

### Ethics Statement

The University of Rochester RSRB has reviewed this study and determined that based on federal (45 CFR 46.102) and University criteria the study does not qualify as human subjects research and has waived the need for consent (RSRB#00024759). All animal procedures were performed under guidelines of the National Institutes of Health and approved by the Institutional Animal Care and Utilization Committee (IACUC) of the University of Rochester Medical Center, Rochester, NY (UCAR#2001–140).

### Lipids

Lipids used in this study were purchased from Matreya and were of highest purity. All lipids were resuspended in anhydrous dimethyl sulfoxide (DMSO) to 10 mM, stored at -20°C or -80°C, and resuspended in media before use. Comparable results for OPC division and survival were obtained with Psy purchased from Sigma-Aldrich and Santa Cruz Biotechnology.

### O-2A/OPC Isolation, Purification, and Culture

Corpus callosa of P7 Sprague-Dawley rats (Charles River) were micro-dissected, finely minced with a sterile blade, and digested for 20 min in 2.2 mg/mL collagenase (Worthington #4189), 20 Kunitz/mL DNase (Sigma D4263) in HBSS (Gibco #114170–161) supplemented with Sato medium (see below). Collagenase-containing medium was then replaced with 20 Kunits/mL DNase and papain solution (1:40, activated per manufacturer’s directions; Worthington #LS003127) in HBSS/Sato for 20 min. Tissue was then sequentially triturated with 21-, 25-, and 26-guage needles in 35 Kunits/mL DNase in DMEM:F12 complete media (see below) before dissociated cells were plated on tissue culture plastic for 10 min (37°C, 7% CO_2_). Nonadherent cells were pelleted by centrifugation (5 min, 500 xg), resuspended in degassed HBSS/Sato supplemented with 1% BSA Fraction V and anti-A2B5 MACS beads (1:50; Miltenyi Biotec #130-093-388), and incubated on ice for 20 min. Cells were pelleted and sorted with MACS columns as per manufacturer’s directions (Miltenyi).

A2B5^+^ cells were plated on tissue culture plastic coated with poly-L-lysine (1 μg/cm^2^ for 20 min; Sigma #P1274) in DMEM:F12 (Gibco #11330–057) supplemented with 10 μg/mL insulin (Sigma #I5500), 100 μg/mL holotransferrin (Sigma #T2252), Sato media (final concentration: 0.03% BSA Fraction V [Sigma #A7979-50ML], 10 μM putrescine [Sigma #P7505], 200 nM progesterone [Sigma #P0130], 235 nM sodium selenite [Sigma #S1382]), 50 μg/mL gentamycin (Gibco #15750–060), 10 ng/mL PDGF-AA (R&D #221-AA), and 5 ng/mL basic FGF (Miltenyi #130–093) and maintained at 37°C (7% CO_2_). Cells were passaged with 0.05% trypsin-EDTA (Gibco #2300), neutralized with 80 Kunitz/mL soybean trypsin inhibitor (Sigma #T9003), and replated in DMEM:F12 complete media supplemented with 10 ng/mL PDGF-AA (and without basic FGF). Purified O-2A/OPCs were passaged no more than once and were maintained in culture for at most 7–9 d in vitro for all experiments. To generate OLs, purified O-2A/OPCs were maintained in DMEM:F12 containing Sato components, transferrin, insulin, 100 pg/mL PDGF-AA, and 45 nM T3/T4 (Sigma #T6397/#0397) for 5 d before initiation of experiments. Human cells were isolated from corpus callosal fields of fetal week 18–21 de-identified tissue, purified with anti-CD140a-coupled magnetic beads (1:100; BD Biosciences #558774; Miltenyi), and maintained as above.

### Cortical and Hippocampal Neuron Isolation and Culture

Embryonic neurons were isolated from E18 Sprague-Dawley rats (Charles River) and maintained as previously described [213]. Briefly, isolated tissue was digested in papain solution (1:50, activated per manufacturer’s directions; Worthington #LS003127) in HBSS/Sato for 20 min at 37°C (7% CO_2_). Pelleted tissue was then triturated with a pulled glass Pasteur pipet in 80 Kunitz/mL DNase (HBSS; Sigma #D4263), and dissociated cells were pelleted through a 0.5 M sucrose cushion (10 min, 500 xg). Immature neurons were plated on poly-L-lysine-coated tissue culture plastic (1 μg/cm^2^ for 20 min; Sigma #P1274) in NeuroBasal media (Gibco #21103–049) with 50 μg/mL gentamycin (Gibco #15750–060), 2 mM L-glutamine (Gibco #25030–081), and 1 X B27 serum-free supplement (Gibco #17504044). Neurons were allowed to mature for 7 d at 37°C (7% CO_2_) before initiation of experiments, with a 50% media change every third day. Analysis of cell survival was determined with calcein-AM/propidium iodide, as described below.

### Clonal Analysis

Purified (A2B5^+^) O-2A/OPCs were isolated from P7 rat corpus callosa as above and plated at a density of 25 cells/cm^2^ in poly-L-lysine-coated 24-well plates in DMEM:F12 complete media supplemented with 10 ng/mL PDGF-AA immediately after purification. Experiments were initiated after 24 h. Cells were fixed after 5 d in 4% paraformaldehyde, stained with antibodies against A2B5 (1:4; in-house hybridoma, ATCC) and GalC (1:4; in-house hybridoma, ATCC), and counterstained with DAPI (1 μg/mL; Invitrogen #D1306), and the size and composition of each clone was then scored. Spontaneous generation of GalC^+^ OLs (i.e., in the absence of differentiation conditions) or Type 2 astrocytes was not detected in this paradigm, and so only the number of progenitor cells (A2B5^+^GalC^−^) per clone is reported.

### Cell Migration

Cell migration with agarose drops was performed as previously reported [214, 215]. Briefly, purified (A2B5^+^) O-2A/OPCs were isolated from P7 rat corpus callosa as above, resuspended in 0.3% low-melt agarose (at 37°C; Sigma #A0701), and diluted in DMEM:F12 complete media supplemented with 10 ng/mL PDGF-AA at a density of 4 x 10^4^ cells/μL, and 1.5 μL of the cell-agarose mixture was plated in the center of a poly-L-lysine-coated 24-well plate. The agarose was allowed to gel at 4°C for 10 min before DMEM:F12 complete media supplemented with 10 ng/mL PDGF-AA, with and without Psy. (PDGF-AA was omitted in some wells as controls for migration, as O-2A/OPC motility is stimulated in vitro by PDGF). Half of the media, with and without Psy, was replaced daily for 3 d, after which point the cells were loaded with calcein-AM (200 nM) and imaged. The distance migrated from the agarose drop to the leading edge was quantified as reported [215].

### Immunocytochemistry

Cells were fixed with 4% paraformaldehyde for 20 min before permeabilization for 10 min with 0.5% Triton X-100 (Sigma #X100) in blocking media (Earl’s Balanced Salt Solution [Gibco] with 5% calf serum and 1% BSA Fraction V). Permeabilized cells were then blocked for 1 h at 25°C before overnight incubation with primary antibodies (A2B5 hybridoma [IgM, 1:4], GalC hybridoma [IgG3, 1:10], Olig2 [1:500; Millipore #MABN50], Ki67 [1:1000, BD Pharmingen #550609], GFAP [1:2000; DAKO #Z0334], Tuj1 [1:2000; Abcam #14545]) diluted in blocking media at 4°C. After washing, cells were incubated with species- and isotype-matched Alexa Fluor-conjugated secondary antibodies (1:2000; Invitrogen) and counterstained with DAPI (1 μg/mL; Invitrogen #D1306) for 30 min at 25°C before final washes with PBS and ddH_2_O.

### Immunohistochemistry

Mice were transcardially perfused with 4% paraformaldehyde/PBS. Isolated tissue was post-fixed for 24 h in 4% paraformaldehyde and normalized for 48 h in 20% sucrose. Brains were sectioned at 15-μm thickness in OCT (Tissue Tek) using a cryotome and immunostained with Ki67 (1:250; BD Pharmingen #550609), Olig2 (1:500; Millipore #MABN50), GST-pi (1:500; BD Biosciences #610718), Fluoromyelin (Invitrogen), and DAPI (Invitrogen). Mosaic images were acquired using a Leica TCS SP5 laser confocal microscope with a 40x oil immersion lens. Data represent analyses of the corpus callosa of at least three WT and three twitcher brains from separate litters.

### Analysis of Cell Survival

Cells were incubated with 200 nM calcein-AM and 1 μg/mL prodium iodide for 30 min at 37°C to determine the proportion of live and dead cells, respectively, in an experimental condition. Single-cell analysis was performed using a Celigo cytometer (Nexcelom) and the %Live corrected values are reported (calcein^+^PI^−^).

### Analysis of Cell Proliferation Rate

The total number of cells per well was determined using Brightfield analysis with a Celigo cytometer (Nexcelom) daily across 5 d, and cell numbers were normalized to the number of cells at the beginning of the experiment (Day 0). The proliferation rate was calculated as the fold change in cell number per unit time (days) using linear regression (0.95 < R^2^ < 1.0 over 5 d) for each well.

### Differentiation

To induce differentiation, purified (A2B5^+^) O-2A/OPCs were exposed to DMEM:F12 complete media supplemented with 1 ng/mL PDGF-AA and 45 nM T3/T4 mixture (Sigma #T6397/#0397) and allowed to differentiate for 5 d. Cells were fixed in 4% paraformaldehyde, stained with antibodies against A2B5 (1:4; in-house hybridoma ATCC) and GalC (1:4; in-house hybridoma, ATCC), and counterstained with DAPI (1 μg/mL; Invitrogen #D1306), and the numbers of GalC^+^ OLs and A2B5^+^GalC^−^ progenitor cells per condition were quantified.

### Small-Molecule Screen

For analyses of cell division, purified (A2B5^+^) O-2A/OPCs were exposed to 1 μM Psy with each of the 1,040 compounds in the NINDS II Custom Collection library (Microsource) diluted to a final concentration of 0.2, 1, and 5 μM, each in duplicate, 15 compounds per plate. Each plate had control wells (in triplicate) of cells exposed to either vehicle (0.01% DMSO) or Psy (1 μM) alone. The proliferation rate was determined by daily cell counting using a Celigo cytometer (Nexcelom) across 5 d, as outlined above, and the increases in cell number within each well were internally normalized to the number of cells in that well at Day 0 (before the addition of Psy/compounds). The calculated proliferation rates were then normalized to the mean proliferation rate of in-plate vehicle-treated controls.

Selected “hits” in both screens (15 for survival and 36 for proliferation) were rescreened at 0.2, 1, and 5 μM for their ability to significantly reduce Psy-induced suppression of division across 5 d. Those compounds that significantly reduced Psy toxicities with at least one of the three selected concentrations (22 in total) were rescreened using nine-point dose-response curves, ranging from 1 nM to 10 μM, to identify optimally protective concentrations to significantly reduce Psy-induced cell death at 5 d and/or suppression of division across 5 d. The list of 22 compounds that significantly reduced Psy-induced suppression of division was shortened to 15 by elimination of those minimally protective compounds for which commercial sources were cost prohibitive or unavailable.

### Growth Factor Screen

For analyses of cell division, cells were exposed to 1 μM Psy with each growth factor (including BSA, with which all growth factors were diluted) diluted to a final concentration of 10, 33, and 100 ng/mL, each in triplicate, six growth factors per plate. Each plate had control wells (in triplicate) of cells exposed to either vehicle (0.01% DMSO) or Psy (1 μM) alone. The proliferation rate was determined by daily cell counting using a Celigo cytometer (Nexcelom) across 5 d, as outlined above, and the increases in cell number within each well were internally normalized to the number of cells in that well at Day 0 (before the addition of Psy/compounds). The calculated proliferation rates were then normalized to the mean proliferation rate of in-plate vehicle-treated controls.

### Fingerprint Analysis

Concentrations of small-molecule inhibitors of signaling proteins were selected based on their ability to reduce phosphorylation of target proteins when analyzed by immunoblot when possible; the selected concentrations minimally enhanced—or did not alter—Psy’s effects on cell division when examined in the absence of protective agents. Purified (A2B5^+^) O-2A/OPCs were pretreated with the small-molecule inhibitors for 1 h before the addition of protective agent and Psy (each in triplicate). All plates had vehicle- and Psy-treated wells (each in triplicate), as well as Psy combined with the protective agent of interest, wells as controls. The proliferation rates were analyzed and normalized against vehicle-treated controls, as outlined above. Data were hierarchically clustered with an unweighted Euclidean distance similarity metric (complete linkage clustering) using Cluster 3.0 and visualized using TreeView.

### Delayed Administration of Protective Agents

For experiments in which the administration of candidate protective agents was delayed, purified (A2B5^+^) O-2A/OPCs were exposed to the indicated concentrations of Psy for 2 d before each small molecule or IGF-1, prepared at a 10X concentration in DMEM:F12 complete media supplemented with 10 ng/mL PDGF-AA, was diluted to 1X so as to minimize dilution of Psy and perturbation of cells. An equal volume of DMEM:F12 complete media supplemented with 10 ng/mL PDGF-AA was added to untreated and Psy-only control wells. The proliferation rate was calculated from the daily change in cell number from time of administration (Day 2) for three days (Day 5), as outlined above.

### Lysosomal pH Measurements

Purified (A2B5^+^) O-2A/OPCs were plated on poly-L-lysine-coated glass-bottom microwell dishes (MatTek Co., Ashland, MA; #P35G-1.5–14) in DMEM:F12 complete media supplemented with 10 ng/mL PDGF-AA after passaging. Cells were loaded with 500 μg/mL LysoSensor Yellow/Blue Dextran (Invitrogen) in complete media with PDGF for 24 h prior to treatment. After 24 h, the cells were fixed in 4% paraformaldehyde and were imaged using a Leica TCS SP5 laser confocal microscope with a 63X oil immersion lens. Using an excitation wavelength of 335 nm (405 diode), emission spectra at 450 nm (acidic) and 521 nm (alkaline) were quantified, and the ratio of these emissions was calculated using the Leica Advanced Fluorescence software. Live-cell imaging was performed as above, with the exception that cells were not fixed in PFA prior to imaging and analysis. To generate the lysosomal pH calibration curve, the pH of pre-loaded O2A/OPCs was measured as previously described [112]. Briefly, the cells were incubated in calibration buffers (20 mM MES, 110 mM KCl, and 20 mM NaCl containing 10 μM monensin and 20 μM nigericin; Sigma) adjusted to known pH values between 4.0 and 6.0 at 0.5 increments using HCl/NaOH for 1 h prior to imaging, and ratiometric quantification, as above. Calibration curves were generated using both fixed and live cells.

### Endocytosis Measurements

Purified (A2B5^+^) O-2A/OPCs were exposed to indicated conditions for 24 h. Cells were trypsinized and resuspended at a density of 10^6^/mL in conditioned (treated) medium and 1:1000 FluoSpheres polystyrene beads (Invitrogen). The cell:bead suspension was incubated at room temperature and gently inverted every 5 min. At indicated time points, 10 μL (10,000 cells) were transferred to 1 mL of ice-cold PBS and pelleted at 13,000 rpm at 4°C for 5 min. Cell pellets were resuspended in ice-cold 2% paraformaldehyde/PBS and transferred to PLL-coated 96-well dishes to adhere during fixation. The integrated fluorescence intensity per cell was measured using a Celigo cytometer (Nexcelom) and plotted over time; the time-to-half-maximal intensity was calculated using curve-fitting software (Prism).

### In Situ Cathepsin Activity Measurements

Cathepsin B activity was measured as per manufacturer’s instructions (MagicRed Cathepsin B substrate, ICT). Briefly, purified (A2B5^+^) O-2A/OPCs were treated as indicated for 24 h before exposure to cell-permeant CathB substrate (1 μM); substrate cleavage occurred at 37°C for 1 h before the integrated fluorescent intensity per cell was quantified with a Celigo cytometer (Nexcelom). For cathepsin D measurements, fluorescently labeled, cell-permeant CathD active-site inhibitor (BODIPY-FL Pepstatin A; 10 μM) was added to O-2A/OPCs that had been treated as indicated for 24 h; active CathD labeling occurred at 37°C for 1 h before the integrated fluorescent intensity per cell was quantified with a Celigo cytometer (Nexcelom).

### In Situ Lipid Accumulation

Neutral lipid and phospholipid accumulation were quantified with the HCS LipidTox Phospholipidosis and Steatosis Detection Kit (Invitrogen) as per the manufacturer’s directions. Positive controls cyclosporin A (10 μM) and propranolol (10 μM) were used, respectively.

### CFTR Knockdown

Rat O-2A/OPCs were exposed to either 50-nM pools of four siRNA constructs targeting rat *CFTR* or 50-nM pools of four control siRNA constructs that do not target the rat genome with DharmaFECT-1 transfection reagent (1:1000), as per manufacturer’s directions (Dharmacon), for 24 h. Four d post transfection, cells were passaged and either lysed for western blot analysis or plated on glass-bottom dishes for analysis of lysosomal pH, as outlined above. Western blot analysis was performed as previously reported [216] using an anti-rabbit CFTR antibody (Cell Signaling) and HRP-conjugated beta-actin (Santa Cruz).

### Psy Quantification

Mice were killed at P35 and transcardially perfused with ice-cold PBS. Isolated tissue was flash-frozen in liquid nitrogen and stored at -80°C until analysis. Analysis of sphingolipids was performed by Dr. Jacek Bielawski from the Lipidomics Core at the Medical University of South Carolina (MUSC) using liquid chromatography-mass spectrometry (LC-MS/MS) and supercritical fluid chromatography-mass spectrometry (SFC-MS/MS) methodologies, as described previously [217].

### Animal Treatment

Adult heterozygote (Galc^*tw*i/+^) C57Bl/6J (B6.CE-Galctwi/J) mice were originally obtained from Dr. Ernesto Bongarzone (University of Illinois at Chicago, Chicago, IL) and used as breeder pairs to generate homozygous (*twi*; *Galc*^*twi*/*twi*^) twitcher mice and WT (Galc^+/+^) C57Bl/6J mice. All animal procedures were approved by the Institutional Animal Care and Use Committee (IACUC) at the University of Rochester School of Medicine and Dentistry and conformed to the requirements of the Animal Welfare Act. In total, three cohorts of aged-matched mice from different litters received daily IP injections beginning at P10: WT mice receiving saline (*n* = 6); *twi* receiving saline (*n* = 6); *twi* receiving 1 mg/kg NKH-477 (Tocris) in saline (*n* = 8). Mice were euthanized at P35 and tissue was isolated after transcardial perfusion with 4% paraformaldehyde. Immunohistochemical analyses were completed as outlined above. For survival analysis, animals were provided moistened chow and hydragel water packs and monitored daily for weight gain. Animals were euthanized when moribund, as assessed by when the animals could no longer ambulate to maintain food and water intake or exhibited clinical signs of pain such as hunched posture and ruffled fur, as determined on a daily basis. Animals were euthanized using CO_2_ exposure and cervical dislocation.

### Motor Behavior Testing

Aged-matched mice from different litters received daily IP injections beginning at P10: WT mice receiving saline (*n* = 4); *twi* receiving saline (*n* = 3); *twi* receiving 1 mg/kg NKH-477 (Tocris) in saline (*n* = 4) were analyzed for locomotive ability and gait using the Phenoscan suite and Runwayscan software (CleverSys, Inc) at P25. Multiple parameters, including stance, stride, swing, brake, and propulsion time (milliseconds), stride length (millimeters), and average speed (millimeters/s) were collected for each animal over three compliant trials and averaged for both front and rear paws.

### Statistical Analyses

Bar graphs are plotted as mean ± SEM and represent at minimum three independent biological replicates performed in triplicate, except where noted. Two-group comparisons were analyzed using a Student’s *t* test, and multiple-group comparisons were analyzed using an ANOVA with Bonferroni post-hoc test. Prism (v5.0; GraphPad) was used for data analysis and presentation.

## Supporting Information

S1 Data Quantification and analyses underlying the data summarized in all figures and Supporting Information figures. (XLSX)

S1 Fig Psy causes a diverse array of cellular and biochemical toxicities in O-2A/OPCs in vitro and in vivo. **(A)** Quantification of the percentage of GalC^+^ rat OLs derived from A2B5^+^ OPCs over 5 d in differentiation conditions, with and without 1 μM Psy. **(B)** Representative immunofluorescent images of rat O-2A/OPCs exposed to 1 μM Psy or vehicle (DMSO) for 1 d, showing cytoskeletal collapse, and stained with phalloidin (actin) and tubulin. **(C)** Representative images and quantification of rat O-2A/OPCs, with and without 3 μM Psy, migrating radially from an agarose drop after 3 d; calcein-AM (green) and prodium iodide (red) were used to identify live and dead cells, respectively. Note that PDGF stimulates O-2A/OPC migration. White dashed line: agarose drop border. **(D)** Representative immunofluorescent images of rat O-2A/OPCs exposed to 1 μM Psy or positive assay controls cyclosporine A (CysA; 10 μM) and propranolol (10 μM) for 2 d, stained for neutral lipid and phospholipid accumulation, respectively. **(E)** Representative brightfield and immunofluorescent images of rat O-2A/OPCs exposed to 1 μM Psy or vehicle (DMSO) for 1 d before the addition of fluorescently labeled nanobeads for the indicated times. **(F)** Quantification of lysosomal pH in live rat O-2A/OPCs exposed to vehicle (0.01% DMSO), 100 nM BafA, or 1 μM Psy for 24 h. **(G)** Representative immunofluorescent time-lapse images of rat O-2A/OPCs exposed to vehicle (0.01% DMSO), 100 nM BafA, or 1 μM Psy for 0–5 min. Data for all graphs displayed as mean ± SEM; *p < 0.05, ^†^*p* < 0.001 versus control, unless otherwise indicated. See also S1, S2 and S3 Movies for time-lapse movies of lysosomal pH changes. Data presented in this figure can be found in S1 Data.
(TIFF)

S2 Fig Unbiased screening identifies chemically diverse candidate protective agents that reduce Psy toxicities. **(A)** Physicochemical characterization of small molecules that reduce Psy-induced **(D)** cell death or **(E)** suppression of division, including atomic composition (% by mass), molecular weight (Daltons), logP partition coefficient, number of ring structures, and surface area (Å^2^). **(B)** Quantification of cell division of rat O-2A/OPCs exposed to 1.5 μM Psy for 5 d, with and without the indicated growth factors at 10, 33, or 100 ng/mL. Data for all graphs displayed as mean ± SEM; ^a^*p* < 0.05, ^b^*p* < 0.01, ^c^*p* < 0.001 versus Psy-only treatment. See S1 and S2 Tables for drugs and concentrations used. Data presented in this figure can be found in S1 Data.
(TIFF)

S3 Fig Protective agents converge on a limited number of common necessary pathways for their activity. Representative “fingerprints of protection” for the functionally and structurally unrelated candidate drugs 2G08, 2F11, and 8D08. Data represent mean ± SEM. See also See S1 and S2 Tables for drugs and concentrations, and S3 Table for details on the “fingerprinting” screen. Data presented in this figure can be found in S1 Data.
(TIFF)

S4 Fig Candidate protective agents do not reduce basal lysosomal pH in the absence of Psy. **(A)** A representative western blot of *CFTR* knockdown versus NT controls in rat O-2A/OPCs, 4 d post transfection. Quantification of lysosomal pH in rat O-2A/OPCs, with or without *CFTR* knockdown (5 d post transfection), exposed to 1 μM Psy or 1 μM Psy and 333 nM RP-107 for 24 h. **(B)** Quantification of lysosomal pH of rat O-2A/OPCs exposed to the indicated drugs for 24 h in the absence of Psy. Data for all graphs displayed as mean ± SEM; *p < 0.05, **p < 0.01, ^†^*p* < 0.001. See S1 and S2 Tables for drugs and concentrations used. Data presented in this figure can be found in S1 Data.
(TIFF)

S5 Fig Protective agents rescue critical O-2A/OPC behaviors and lysosomal function in response to lysosphingolipids accumulating in other LSDs. **(A)** Proliferation analysis of rat O-2A/OPCs exposed to 1.5 μM Psy, 1 μM GlcSph, 3 μM Lyso-SF, or 12 μM LacSph for 5 d, with and without the indicated protective agents. **(B)** Proliferation analysis of rat O-2A/OPCs exposed to 1.5 μM Psy, 1 μM GlcSph, 3 μM Lyso-SF, or 12 μM LacSph for 5 d, with and without the indicated protective agents, which were administered 2 d after the indicated lyso-lipid. **(C)** Venn diagram summarizing **(B)** for all lyso-lipids. Data for all graphs displayed as mean ± SEM; ^a^*p* < 0.05, ^b^*p* < 0.01, ^c^*p* < 0.001 versus lipid-only treatment. See S1 and S2 Tables for drugs and concentrations used. Data presented in this figure can be found in S1 Data.
(TIFF)

S6 Fig Lysosphingolipids disrupt human O-2A/OPC behaviors. **(A)** Representative immunofluorescent images of human fetal O-2A/OPCs maintained in 10 ng/mL PDGF + 10 ng/mL bFGF (“PDGF+FGF”); 100 pg/mL PDGF (“- PDGF”); 1 ng/mL PDGF + 40 ng/mL T3/T4 (“+T3/T4”); and 1 ng/mL PDGF + 10 ng/mL BMP4 (“+BMP4”) for 5 d in mass culture. A2B5^+^: glial progenitor cells; GalC^+^: OLs; GFAP: astrocytes; A2B5^+^/GFAP^+^: Type-2 astrocytes; Ki67^+^: mitotically active cells; Olig2^+^: oligodendroglial-lineage cells. **(B)** Quantification of **(A)**. **(C)** Quantification of human fetal O-2A/OPCs maintained as in **(A)** for 5 d, except at clonal density. The mean number of cells immunopositive for the indicated stain per clone, as well as the percentage of clones containing at least one immunopositive cell, are reported. Note that NeuN^+^ or Tuj1^+^ neurons were never detected in mass or clonal culture. Data for all graphs displayed as mean ± SD for one GW20 human sample. All experiments were repeated in four human GW19-21 samples with comparable results. Data presented in this figure can be found in S1 Data.
(TIFF)

S7 Fig NKH-477 treatment reduces *twitcher* mouse gait abnormalities. Quantification of gait for P25 vehicle-treated WT (*n* = 3–4), vehicle-treated *twitcher* mice (*n* = 3), and NKH-treated *twitcher* mice (*n* = 4), including measurements of stance, break, propel, swing, and stride time, as well stride length, for front and rear paws. Data for all graphs displayed as mean ± SEM; *p < 0.05, **p < 0.01, ^†^*p* < 0.001 versus WT; ^a^*p* < 0.05, ^c^*p* < 0.001 versus vehicle-treated *twitcher*. Data presented in this figure can be found in S1 Data.
(TIFF)

S1 Movie Time-lapse movie of rat O-2A/OPCs loaded with a ratiometric lysosomal pH dye and exposed to vehicle (0.01% DMSO) for 5 min. (MP4)

S2 Movie Time-lapse movie of rat O-2A/OPCs loaded with a ratiometric lysosomal pH dye and exposed to 100 nM BafA for 5 min. (MP4)

S3 Movie Time-lapse movie of rat O-2A/OPCs loaded with a ratiometric lysosomal pH dye and exposed to 1 μM Psy for 5 min. (MP4)

S1 Table Chemical structures of lead protective compounds. (DOCX)

S2 Table List of lead protective compounds, optimal concentrations used in human and rat O-2A/OPCs, and meta-analyses of clinical usage. (DOCX)

S3 Table List of pharmacological inhibitors, their concentrations, and their protein targets used in the fingerprinting secondary screen. (DOCX)

S4 Table List of lipid concentrations used in human and rat O-2A/OPCs. (DOCX)

## Abbreviations

BafA: bafilomycin A
BODIPY: boron-dipyrromethene
cAMP: cyclic AMP
CFTR: cystic fibrosis transmembrane conductance regulator
CFTRi-172: CFTR-inhibitor 172
CNS: central nervous system
EIKD: early infantile KD
FDA: United States Food and Drug Administration
GALC: galactocerebrosidase
GGT: geranylgeranyl transferase
GlcCer: glucosylceramide
GlcSph: glucosylsphingosine
IGF-1: insulin-like growth factor
IP: intraperitoneal
JNK: Jun N-terminal kinase
KD: Krabbe disease
LacSer: lactosylceramide
LacSph: lactosylsphingosine
LSDs: lysosomal storage disorders
lyso-SF: lyso-sulfatide
MAPK: mitogen-activated protein kinase
MLD: metachromatic leukodystrophy
mTOR: mammalian target of rapamycin
N-AcPsy: N-acetyl-Psy
N-AcSF: N-acetyl-sulfatide
NBS: newborn screening
NT: nontargeting
O-2A/OPCs: OL/type-2 astrocyte progenitor cells
OL: oligodendrocyte
P: postnatal day
PI3K: phosphoinositide-3-kinase
PKA: protein kinase A
PKC: protein kinase C
PNS: peripheral nervous systems
Psy: psychosine
RAF: rapidly accelerated fibrosarcoma gene
SEM: standard error of the mean
siRNA: small interfering RNA
WT: wild-type

## References

[1] Reddy AS, Kim JH, Hawkins-Salsbury JA, Macauley SL, Tracy ET, Vogler CA, et al Bone marrow transplantation augments the effect of brain- and spinal cord-directed adeno-associated virus 2/5 gene therapy by altering inflammation in the murine model of globoid-cell leukodystrophy. J Neurosci. 2011;31(27):9945–57. 10.1523/JNEUROSCI.1802-11.2011 21734286 PMC3348856

[2] Lin D, Donsante A, Macauley S, Levy B, Vogler C, Sands MS. Central nervous system-directed AAV2/5-mediated gene therapy synergizes with bone marrow transplantation in the murine model of globoid-cell leukodystrophy. Mol Ther. 2007;15(1):44–52. 10.1038/sj.mt.6300026 17164774

[3] Gentner B, Visigalli I, Hiramatsu H, Lechman E, Ungari S, Giustacchini A, et al Identification of hematopoietic stem cell-specific miRNAs enables gene therapy of globoid cell leukodystrophy. Sci Transl Med. 2010;2(58):58ra84 10.1126/scitranslmed.3001522 21084719

[4] Ripoll CB, Flaat M, Klopf-Eiermann J, Fisher-Perkins JM, Trygg CB, Scruggs BA, et al Mesenchymal lineage stem cells have pronounced anti-inflammatory effects in the twitcher mouse model of Krabbe's disease. Stem Cells. 2011;29(1):67–77. 10.1002/stem.555 21280158 PMC3412284

[5] Wicks SE, Londot H, Zhang B, Dowden J, Klopf-Eiermann J, Fisher-Perkins JM, et al Effect of intrastriatal mesenchymal stromal cell injection on progression of a murine model of Krabbe disease. Behav Brain Res. 2011;225(2):415–25. 10.1016/j.bbr.2011.07.051 21840342 PMC3179783

[6] Miranda CO, Teixeira CA, Liz MA, Sousa VF, Franquinho F, Forte G, et al Systemic delivery of bone marrow-derived mesenchymal stromal cells diminishes neuropathology in a mouse model of Krabbe's disease. Stem Cells. 2011;29(11):1738–51. 10.1002/stem.724 21898691

[7] Miranda CO, Teixeira CA, Sousa VF, Santos TE, Liz MA, Marques AM, et al Primary bone marrow mesenchymal stromal cells rescue the axonal phenotype of Twitcher mice. Cell Transplant. 2014;23(2):239–52. 10.3727/096368913X669752 23809254

[8] Strazza M, Luddi A, Carbone M, Rafi MA, Costantino-Ceccarini E, Wenger DA. Significant correction of pathology in brains of twitcher mice following injection of genetically modified mouse neural progenitor cells. Mol Genet Metab. 2009;97(1):27–34. 10.1016/j.ymgme.2009.01.005 19217332

[9] Lee WC, Tsoi YK, Troendle FJ, DeLucia MW, Ahmed Z, Dicky CA, et al Single-dose intracerebroventricular administration of galactocerebrosidase improves survival in a mouse model of globoid cell leukodystrophy. FASEB J. 2007;21(10):2520–7. 10.1096/fj.06-6169com 17403939

[10] Qin EY, Hawkins-Salsbury JA, Jiang X, Reddy AS, Farber NB, Ory DS, et al Bone marrow transplantation increases efficacy of central nervous system-directed enzyme replacement therapy in the murine model of globoid cell leukodystrophy. Mol Genet Metab. 2012;107(1–2):186–96. 10.1016/j.ymgme.2012.05.021 22704480 PMC3444533

[11] Lattanzi A, Neri M, Maderna C, di Girolamo I, Martino S, Orlacchio A, et al Widespread enzymatic correction of CNS tissues by a single intracerebral injection of therapeutic lentiviral vector in leukodystrophy mouse models. Hum Mol Genet. 2010;19(11):2208–27. 10.1093/hmg/ddq099 20203170

[12] Lattanzi A, Salvagno C, Maderna C, Benedicenti F, Morena F, Kulik W, et al Therapeutic benefit of lentiviral-mediated neonatal intracerebral gene therapy in a mouse model of globoid cell leukodystrophy. Hum Mol Genet. 2014;23(12):3250–68. 10.1093/hmg/ddu034 24463623 PMC4030779

[13] Shen JS, Watabe K, Ohashi T, Eto Y. Intraventricular administration of recombinant adenovirus to neonatal twitcher mouse leads to clinicopathological improvements. Gene Ther. 2001;8(14):1081–7. 10.1038/sj.gt.3301495 11526455

[14] Shen JS, Meng XL, Yokoo T, Sakurai K, Watabe K, Ohashi T, et al Widespread and highly persistent gene transfer to the CNS by retrovirus vector in utero: implication for gene therapy to Krabbe disease. J Gene Med. 2005;7(5):540–51. 10.1002/jgm.719 15685691

[15] Lin DS, Hsiao CD, Liau I, Lin SP, Chiang MF, Chuang CK, et al CNS-targeted AAV5 gene transfer results in global dispersal of vector and prevention of morphological and function deterioration in CNS of globoid cell leukodystrophy mouse model. Mol Genet Metab. 2011;103(4):367–77. 10.1016/j.ymgme.2011.05.005 21620749

[16] Rafi MA, Zhi Rao H, Passini MA, Curtis M, Vanier MT, Zaka M, et al AAV-mediated expression of galactocerebrosidase in brain results in attenuated symptoms and extended life span in murine models of globoid cell leukodystrophy. Mol Ther. 2005;11(5):734–44. 10.1016/j.ymthe.2004.12.020 15851012

[17] LeVine SM, Pedchenko TV, Bronshteyn IG, Pinson DM. L-cycloserine slows the clinical and pathological course in mice with globoid cell leukodystrophy (twitcher mice). J Neurosci Res. 2000;60(2):231–6. 10.1002/(SICI)1097-4547(20000415)60:2<231::AID-JNR12>3.0.CO;2-E 10740228

[18] Parenti G, Pignata C, Vajro P, Salerno M. New strategies for the treatment of lysosomal storage diseases (review). Int J Mol Med. 2013;31(1):11–20. 10.3892/ijmm.2012.1187 23165354

[19] Ortolano S, Viéitez I, Navarro C, Spuch C. Treatment of lysosomal storage diseases: recent patents and future strategies. Recent Pat Endocr Metab Immune Drug Discov. 2014;8(1):9–25. 24433521 10.2174/1872214808666140115111350

[20] van Gelder CM, Vollebregt AA, Plug I, van der Ploeg AT, Reuser AJ. Treatment options for lysosomal storage disorders: developing insights. Expert Opin Pharmacother. 2012;13(16):2281–99. 10.1517/14656566.2012.729039 23009070

[21] Desnick RJ, Schuchman EH. Enzyme replacement therapy for lysosomal diseases: lessons from 20 years of experience and remaining challenges. Annu Rev Genomics Hum Genet. 2012;13:307–35. 10.1146/annurev-genom-090711-163739 22970722

[22] Gordon HB, Letsou A, Bonkowsky JL. The leukodystrophies. Semin Neurol. 2014;34(3):312–20. 10.1055/s-0034-1386769 25192509

[23] Cox TM. Eliglustat tartrate, an orally active glucocerebroside synthase inhibitor for the potential treatment of Gaucher disease and other lysosomal storage diseases. Curr Opin Investig Drugs. 2010;11(10):1169–81. 20872320

[24] Butters TD, Dwek RA, Platt FM. New therapeutics for the treatment of glycosphingolipid lysosomal storage diseases. Adv Exp Med Biol. 2003;535:219–26. 14714898 10.1007/978-1-4615-0065-0_14

[25] Barranger JM, Novelli EA. Gene therapy for lysosomal storage disorders. Expert Opin Biol Ther. 2001;1(5):857–67. 10.1517/14712598.1.5.857 11728220

[26] Wenger DA, Luzi P, Rafi MA. Lysosomal storage diseases: heterogeneous group of disorders. Bioimpacts. 2013;3(4):145–7. 10.5681/bi.2013.029 24455477 PMC3892733

[27] Eto Y, Shen JS, Meng XL, Ohashi T. Treatment of lysosomal storage disorders: cell therapy and gene therapy. J Inherit Metab Dis. 2004;27(3):411–5. 10.1023/B:BOLI.0000031170.69676.68 15190197

[28] Parenti G, Andria G, Ballabio A. Lysosomal storage diseases: from pathophysiology to therapy. Annu Rev Med. 2015;66:471–86. 10.1146/annurev-med-122313-085916 25587658

[29] Hollak CE, Wijburg FA. Treatment of lysosomal storage disorders: successes and challenges. J Inherit Metab Dis. 2014;37(4):587–98. 10.1007/s10545-014-9718-3 24820227

[30] Platt FM, Boland B, van der Spoel AC. The cell biology of disease: lysosomal storage disorders: the cellular impact of lysosomal dysfunction. J Cell Biol. 2012;199(5):723–34. 10.1083/jcb.201208152 23185029 PMC3514785

[31] Platt FM. Sphingolipid lysosomal storage disorders. Nature. 2014;510(7503):68–75. 10.1038/nature13476 24899306

[32] Cox TM, Cachon-Gonzalez MB. The cellular pathology of lysosomal diseases. J Pathol. 2012;226(2):241–54. 10.1002/path.3021 21990005

[33] Rastall DP, Amalfitano A. Recent advances in gene therapy for lysosomal storage disorders. Appl Clin Genet. 2015;8:157–69. 10.2147/TACG.S57682 26170711 PMC4485851

[34] Weinreb NJ. Oral small molecule therapy for lysosomal storage diseases. Pediatr Endocrinol Rev. 2013;11 Suppl 1:77–90. 24380126

[35] Jakobkiewicz-Banecka J, Wegrzyn A, Wegrzyn G. Substrate deprivation therapy: a new hope for patients suffering from neuronopathic forms of inherited lysosomal storage diseases. J Appl Genet. 2007;48(4):383–8. 10.1007/BF03195237 17998597

[36] Scarpa M, Bellettato CM, Lampe C, Begley DJ. Neuronopathic lysosomal storage disorders: Approaches to treat the central nervous system. Best Pract Res Clin Endocrinol Metab. 2015;29(2):159–71. 10.1016/j.beem.2014.12.001 25987170

[37] Boyd RE, Valenzano KJ. Correction of lysosomal dysfunction as a therapeutic strategy for neurodegenerative diseases. Bioorg Med Chem Lett. 2014;24(14):3001–5. 10.1016/j.bmcl.2014.04.108 24894562

[38] Boudes PF. Clinical studies in lysosomal storage diseases: past, present and future. Pediatr Endocrinol Rev. 2013;11 Suppl 1:68–76. 24380125

[39] Prada CE, Grabowski GA. Neuronopathic lysosomal storage diseases: clinical and pathologic findings. Dev Disabil Res Rev. 2013;17(3):226–46. 10.1002/ddrr.1116 23798011

[40] Hawkins-Salsbury JA, Shea L, Jiang X, Hunter DA, Guzman AM, Reddy AS, et al Mechanism-based combination treatment dramatically increases therapeutic efficacy in murine globoid cell leukodystrophy. J Neurosci. 2015;35(16):6495–505. 10.1523/JNEUROSCI.4199-14.2015 25904800 PMC4405559

[41] Weinreb NJ, Charrow J, Andersson HC, Kaplan P, Kolodny EH, Mistry P, et al Effectiveness of enzyme replacement therapy in 1028 patients with type 1 Gaucher disease after 2 to 5 years of treatment: a report from the Gaucher Registry. Am J Med. 2002;113(2):112–9. 12133749 10.1016/s0002-9343(02)01150-6

[42] Schiffmann R, Kopp JB, Austin HA, Sabnis S, Moore DF, Weibel T, et al Enzyme replacement therapy in Fabry disease: a randomized controlled trial. JAMA. 2001;285(21):2743–9. 11386930 10.1001/jama.285.21.2743

[43] Schiffmann R, Floeter MK, Dambrosia JM, Gupta S, Moore DF, Sharabi Y, et al Enzyme replacement therapy improves peripheral nerve and sweat function in Fabry disease. Muscle Nerve. 2003;28(6):703–10. 10.1002/mus.10497 14639584

[44] Ohashi T. Enzyme replacement therapy for lysosomal storage diseases. Pediatr Endocrinol Rev. 2012;10 Suppl 1:26–34. 23330243

[45] Matthes F, Andersson C, Stein A, Eistrup C, Fogh J, Gieselmann V, et al Enzyme replacement therapy of a novel humanized mouse model of globoid cell leukodystrophy. Exp Neurol. 2015;271:36–45. 10.1016/j.expneurol.2015.04.020 25956830

[46] Fukuda T, Sugie H. [Enzyme Replacement Therapy for Pompe Disease: The Long-Term Efficacy and Limitation]. Brain Nerve. 2015;67(9):1091–8. 10.11477/mf.1416200265 26329149

[47] Nilsson O, Svennerholm L. Accumulation of glucosylceramide and glucosylsphingosine (psychosine) in cerebrum and cerebellum in infantile and juvenile Gaucher disease. J Neurochem. 1982;39(3):709–18. 7097276 10.1111/j.1471-4159.1982.tb07950.x

[48] Toda K, Kobayashi T, Goto I, Ohno K, Eto Y, Inui K, et al Lysosulfatide (sulfogalactosylsphingosine) accumulation in tissues from patients with metachromatic leukodystrophy. J Neurochem. 1990;55(5):1585–91. 1976756 10.1111/j.1471-4159.1990.tb04942.x

[49] Toda K, Kobayashi T, Goto I, Kurokawa T, Ogomori K. Accumulation of lysosulfatide (sulfogalactosylsphingosine) in tissues of a boy with metachromatic leukodystrophy. Biochem Biophys Res Commun. 1989;159(2):605–11. 2539117 10.1016/0006-291x(89)90037-5

[50] Sasagasako N, Kobayashi T, Yamaguchi Y, Shinnoh N, Goto I. Glucosylceramide and glucosylsphingosine metabolism in cultured fibroblasts deficient in acid beta-glucosidase activity. J Biochem. 1994;115(1):113–9. 8188616 10.1093/oxfordjournals.jbchem.a124284

[51] Hannun YA, Bell RM. Lysosphingolipids inhibit protein kinase C: implications for the sphingolipidoses. Science. 1987;235(4789):670–4. 3101176 10.1126/science.3101176

[52] Atsumi S, Nosaka C, Iinuma H, Umezawa K. Accumulation of tissue glucosylsphingosine in Gaucher-like mouse induced by the glucosylceramidase inhibitor cyclophellitol. Arch Biochem Biophys. 1993;304(1):302–4. 10.1006/abbi.1993.1353 8323295

[53] Kohlschütter A. Lysosomal leukodystrophies: Krabbe disease and metachromatic leukodystrophy. Handb Clin Neurol. 2013;113:1611–8. 10.1016/B978-0-444-59565-2.00029-0 23622382

[54] Thekkedath R, Koshkaryev A, Torchilin VP. Lysosome-targeted octadecyl-rhodamine B-liposomes enhance lysosomal accumulation of glucocerebrosidase in Gaucher's cells in vitro. Nanomedicine (Lond). 2013;8(7):1055–65. 23199221 10.2217/nnm.12.138 PMC3644353

[55] Prence EM, Chaturvedi P, Newburg DS. In vitro accumulation of glucocerebroside in neuroblastoma cells: a model for study of Gaucher disease pathobiology. J Neurosci Res. 1996;43(3):365–71. 10.1002/(SICI)1097-4547(19960201)43:3<365::AID-JNR11>3.0.CO;2-4 8714525

[56] Miyatake T, Suzuki K. Globoid cell leukodystrophy: additional deficiency of psychosine galactosidase. Biochem Biophys Res Commun. 1972;48(3):539–43. 5047687 10.1016/0006-291x(72)90381-6

[57] Miyatake T, Suzuki K. Additional deficiency of psychosine galactosidase in globoid cell leukodystrophy: an implication to enzyme replacement therapy. Birth Defects Orig Artic Ser. 1973;9(2):136–40. 4611526

[58] Kobayashi T, Shinoda H, Goto I, Yamanaka T, Suzuki Y. Globoid cell leukodystrophy is a generalized galactosylsphingosine (psychosine) storage disease. Biochem Biophys Res Commun. 1987;144(1):41–6. 3579916 10.1016/s0006-291x(87)80472-2

[59] Suzuki K. Twenty five years of the "psychosine hypothesis": a personal perspective of its history and present status. Neurochem Res. 1998;23(3):251–9. 9482237 10.1023/a:1022436928925

[60] Suzuki K. Globoid cell leukodystrophy (Krabbe's disease): update. J Child Neurol. 2003;18(9):595–603. 14572137 10.1177/08830738030180090201

[61] Voccoli V, Tonazzini I, Signore G, Caleo M, Cecchini M. Role of extracellular calcium and mitochondrial oxygen species in psychosine-induced oligodendrocyte cell death. Cell Death Dis. 2014;5:e1529 10.1038/cddis.2014.483 25412308 PMC4260741

[62] Khan M, Haq E, Giri S, Singh I, Singh AK. Peroxisomal participation in psychosine-mediated toxicity: implications for Krabbe's disease. J Neurosci Res. 2005;80(6):845–54. 10.1002/jnr.20529 15898099

[63] Hawkins-Salsbury JA, Parameswar AR, Jiang X, Schlesinger PH, Bongarzone E, Ory DS, et al Psychosine, the cytotoxic sphingolipid that accumulates in globoid cell leukodystrophy, alters membrane architecture. J Lipid Res. 2013;54(12):3303–11. 10.1194/jlr.M039610 24006512 PMC3826678

[64] White AB, Givogri MI, Lopez-Rosas A, Cao H, van Breemen R, Thinakaran G, et al Psychosine accumulates in membrane microdomains in the brain of krabbe patients, disrupting the raft architecture. J Neurosci. 2009;29(19):6068–77. 10.1523/JNEUROSCI.5597-08.2009 19439584 PMC6665501

[65] Smith B, Galbiati F, Castelvetri LC, Givogri MI, Lopez-Rosas A, Bongarzone ER. Peripheral neuropathy in the Twitcher mouse involves the activation of axonal caspase 3. ASN Neuro. 2011;3(4). 10.1042/AN20110019 PMC3192484 21929508

[66] Cantuti-Castelvetri L, Zhu H, Givogri MI, Chidavaenzi RL, Lopez-Rosas A, Bongarzone ER. Psychosine induces the dephosphorylation of neurofilaments by deregulation of PP1 and PP2A phosphatases. Neurobiol Dis. 2012;46(2):325–35. 10.1016/j.nbd.2012.01.013 22326830 PMC3323754

[67] Cantuti Castelvetri L, Givogri MI, Hebert A, Smith B, Song Y, Kaminska A, et al The sphingolipid psychosine inhibits fast axonal transport in Krabbe disease by activation of GSK3β and deregulation of molecular motors. J Neurosci. 2013;33(24):10048–56. 10.1523/JNEUROSCI.0217-13.2013 23761900 PMC3682375

[68] Cantuti-Castelvetri L, Maravilla E, Marshall M, Tamayo T, D'auria L, Monge J, et al Mechanism of neuromuscular dysfunction in Krabbe disease. J Neurosci. 2015;35(4):1606–16. 10.1523/JNEUROSCI.2431-14.2015 25632136 PMC4308604

[69] Giri S, Jatana M, Rattan R, Won JS, Singh I, Singh AK. Galactosylsphingosine (psychosine)-induced expression of cytokine-mediated inducible nitric oxide synthases via AP-1 and C/EBP: implications for Krabbe disease. FASEB J. 2002;16(7):661–72. 10.1096/fj.01-0798com 11978730

[70] Giri S, Khan M, Rattan R, Singh I, Singh AK. Krabbe disease: psychosine-mediated activation of phospholipase A2 in oligodendrocyte cell death. J Lipid Res. 2006;47(7):1478–92. 10.1194/jlr.M600084-JLR200 16645197

[71] Giri S, Khan M, Nath N, Singh I, Singh AK. The role of AMPK in psychosine mediated effects on oligodendrocytes and astrocytes: implication for Krabbe disease. J Neurochem. 2008;105(5):1820–33. 10.1111/j.1471-4159.2008.05279.x 18248608 PMC2673995

[72] Haq E, Contreras MA, Giri S, Singh I, Singh AK. Dysfunction of peroxisomes in twitcher mice brain: a possible mechanism of psychosine-induced disease. Biochem Biophys Res Commun. 2006;343(1):229–38. 10.1016/j.bbrc.2006.02.131 16530726

[73] Zaka M, Wenger DA. Psychosine-induced apoptosis in a mouse oligodendrocyte progenitor cell line is mediated by caspase activation. Neurosci Lett. 2004;358(3):205–9. 10.1016/j.neulet.2003.12.126 15039117

[74] Haq E, Giri S, Singh I, Singh AK. Molecular mechanism of psychosine-induced cell death in human oligodendrocyte cell line. J Neurochem. 2003;86(6):1428–40. 12950451 10.1046/j.1471-4159.2003.01941.x

[75] Ijichi K, Brown GD, Moore CS, Lee JP, Winokur PN, Pagarigan R, et al MMP-3 mediates psychosine-induced globoid cell formation: implications for leukodystrophy pathology. Glia. 2013;61(5):765–77. 10.1002/glia.22471 23404611 PMC3804069

[76] Duchen LW, Eicher EM, Jacobs JM, Scaravilli F, Teixeira F. Hereditary leucodystrophy in the mouse: the new mutant twitcher. Brain. 1980;103(3):695–710. 7417782 10.1093/brain/103.3.695

[77] Whitfield PD, Sharp PC, Taylor R, Meikle P. Quantification of galactosylsphingosine in the twitcher mouse using electrospray ionization-tandem mass spectrometry. J Lipid Res. 2001;42(12):2092–5. 11734583

[78] Igisu H, Suzuki K. Glycolipids of the spinal cord, sciatic nerve, and systemic organs of the twitcher mouse. J Neuropathol Exp Neurol. 1984;43(1):22–36. 6693925 10.1097/00005072-198401000-00002

[79] Shinoda H, Kobayashi T, Katayama M, Goto I, Nagara H. Accumulation of galactosylsphingosine (psychosine) in the twitcher mouse: determination by HPLC. J Neurochem. 1987;49(1):92–9. 3585345 10.1111/j.1471-4159.1987.tb03399.x

[80] Nozawa M, Iwamoto T, Tokoro T, Eto Y. Novel procedure for measuring psychosine derivatives by an HPLC method. J Neurochem. 1992;59(2):607–9. 1629732 10.1111/j.1471-4159.1992.tb09412.x

[81] Santambrogio S, Ricca A, Maderna C, Ieraci A, Aureli M, Sonnino S, et al The galactocerebrosidase enzyme contributes to maintain a functional neurogenic niche during early post-natal CNS development. Hum Mol Genet. 2012;21(21):4732–50. 10.1093/hmg/dds313 22859505

[82] Zaka M, Rafi MA, Rao HZ, Luzi P, Wenger DA. Insulin-like growth factor-1 provides protection against psychosine-induced apoptosis in cultured mouse oligodendrocyte progenitor cells using primarily the PI3K/Akt pathway. Mol Cell Neurosci. 2005;30(3):398–407. 10.1016/j.mcn.2005.08.004 16169744

[83] Tanaka K, Webster HD. Effects of psychosine (galactosylsphingosine) on the survival and the fine structure of cultured Schwann cells. J Neuropathol Exp Neurol. 1993;52(5):490–8. 8360702 10.1097/00005072-199309000-00007

[84] Svennerholm L, Vanier MT, Månsson JE. Krabbe disease: a galactosylsphingosine (psychosine) lipidosis. J Lipid Res. 1980;21(1):53–64. 7354254

[85] Igisu H, Suzuki K. Progressive accumulation of toxic metabolite in a genetic leukodystrophy. Science. 1984;224(4650):753–5. 6719111 10.1126/science.6719111

[86] Vitner EB, Dekel H, Zigdon H, Shachar T, Farfel-Becker T, Eilam R, et al Altered expression and distribution of cathepsins in neuronopathic forms of Gaucher disease and in other sphingolipidoses. Hum Mol Genet. 2010;19(18):3583–90. 10.1093/hmg/ddq273 20616152

[87] Amritraj A, Peake K, Kodam A, Salio C, Merighi A, Vance JE, et al Increased activity and altered subcellular distribution of lysosomal enzymes determine neuronal vulnerability in Niemann-Pick type C1-deficient mice. Am J Pathol. 2009;175(6):2540–56. 10.2353/ajpath.2009.081096 19893049 PMC2789601

[88] Colletti GA, Miedel MT, Quinn J, Andharia N, Weisz OA, Kiselyov K. Loss of lysosomal ion channel transient receptor potential channel mucolipin-1 (TRPML1) leads to cathepsin B-dependent apoptosis. J Biol Chem. 2012;287(11):8082–91. 10.1074/jbc.M111.285536 22262857 PMC3318733

[89] Tapper H, Sundler R. Bafilomycin A1 inhibits lysosomal, phagosomal, and plasma membrane H(+)-ATPase and induces lysosomal enzyme secretion in macrophages. J Cell Physiol. 1995;163(1):137–44. 10.1002/jcp.1041630116 7896890

[90] Poole B, Ohkuma S. Effect of weak bases on the intralysosomal pH in mouse peritoneal macrophages. J Cell Biol. 1981;90(3):665–9. 6169733 10.1083/jcb.90.3.665 PMC2111912

[91] Heemskerk J, Tobin AJ, Bain LJ. Teaching old drugs new tricks. Meeting of the Neurodegeneration Drug Screening Consortium, 7–8 April 2002, Washington, DC, USA. Trends Neurosci. 2002;25(10):494–6. 12220871 10.1016/s0166-2236(02)02236-1

[92] Cho KH, Kim MW, Kim SU. Tissue culture model of Krabbe's disease: psychosine cytotoxicity in rat oligodendrocyte culture. Dev Neurosci. 1997;19(4):321–7. 9215877 10.1159/000111228

[93] Yamada H, Martin P, Suzuki K. Impairment of protein kinase C activity in twitcher Schwann cells in vitro. Brain Res. 1996;718(1–2):138–44. 8773776 10.1016/0006-8993(96)00098-4

[94] White AB, Galbiati F, Givogri MI, Lopez Rosas A, Qiu X, van Breemen R, et al Persistence of psychosine in brain lipid rafts is a limiting factor in the therapeutic recovery of a mouse model for Krabbe disease. J Neurosci Res. 2011;89(3):352–64. 10.1002/jnr.22564 21259322 PMC3064524

[95] Yamada H, Suzuki K. Responses to cyclic AMP is impaired in the twitcher Schwann cells in vitro. Brain Res. 1999;816(2):390–5. 9878848 10.1016/s0006-8993(98)01142-1

[96] Bibollet-Bahena O, Almazan G. IGF-1-stimulated protein synthesis in oligodendrocyte progenitors requires PI3K/mTOR/Akt and MEK/ERK pathways. J Neurochem. 2009;109(5):1440–51. 10.1111/j.1471-4159.2009.06071.x 19453943

[97] Narayanan SP, Flores AI, Wang F, Macklin WB. Akt signals through the mammalian target of rapamycin pathway to regulate CNS myelination. J Neurosci. 2009;29(21):6860–70. 10.1523/JNEUROSCI.0232-09.2009 19474313 PMC2757755

[98] Tyler WA, Jain MR, Cifelli SE, Li Q, Ku L, Feng Y, et al Proteomic identification of novel targets regulated by the mammalian target of rapamycin pathway during oligodendrocyte differentiation. Glia. 2011;59(11):1754–69. 10.1002/glia.21221 21858874 PMC3174285

[99] Tiwari-Woodruff S, Morales LB, Lee R, Voskuhl RR. Differential neuroprotective and antiinflammatory effects of estrogen receptor (ER)alpha and ERbeta ligand treatment. Proc Natl Acad Sci U S A. 2007;104(37):14813–8. 10.1073/pnas.0703783104 17785421 PMC1976208

[100] Morales LB, Loo KK, Liu HB, Peterson C, Tiwari-Woodruff S, Voskuhl RR. Treatment with an estrogen receptor alpha ligand is neuroprotective in experimental autoimmune encephalomyelitis. J Neurosci. 2006;26(25):6823–33. 10.1523/JNEUROSCI.0453-06.2006 16793889 PMC6673842

[101] Kumar S, Patel R, Moore S, Crawford DK, Suwanna N, Mangiardi M, et al Estrogen receptor β ligand therapy activates PI3K/Akt/mTOR signaling in oligodendrocytes and promotes remyelination in a mouse model of multiple sclerosis. Neurobiol Dis. 2013;56:131–44. 10.1016/j.nbd.2013.04.005 23603111 PMC3674189

[102] Casey JR, Grinstein S, Orlowski J. Sensors and regulators of intracellular pH. Nat Rev Mol Cell Biol. 2010;11(1):50–61. 10.1038/nrm2820 19997129

[103] Wong CO, Li R, Montell C, Venkatachalam K. Drosophila TRPML is required for TORC1 activation. Curr Biol. 2012;22(17):1616–21. 10.1016/j.cub.2012.06.055 22863314 PMC3443270

[104] Luzio JP, Bright NA, Pryor PR. The role of calcium and other ions in sorting and delivery in the late endocytic pathway. Biochem Soc Trans. 2007;35(Pt 5):1088–91. 10.1042/BST0351088 17956286

[105] Cang C, Bekele B, Ren D. The voltage-gated sodium channel TPC1 confers endolysosomal excitability. Nat Chem Biol. 2014;10(6):463–9. 10.1038/nchembio.1522 24776928

[106] Cang C, Aranda K, Seo YJ, Gasnier B, Ren D. TMEM175 Is an Organelle K(+) Channel Regulating Lysosomal Function. Cell. 2015;162(5):1101–12. 10.1016/j.cell.2015.08.002 26317472

[107] Kasper D, Planells-Cases R, Fuhrmann JC, Scheel O, Zeitz O, Ruether K, et al Loss of the chloride channel ClC-7 leads to lysosomal storage disease and neurodegeneration. EMBO J. 2005;24(5):1079–91. 10.1038/sj.emboj.7600576 15706348 PMC554126

[108] Lange PF, Wartosch L, Jentsch TJ, Fuhrmann JC. ClC-7 requires Ostm1 as a beta-subunit to support bone resorption and lysosomal function. Nature. 2006;440(7081):220–3. 10.1038/nature04535 16525474

[109] Graves AR, Curran PK, Smith CL, Mindell JA. The Cl-/H+ antiporter ClC-7 is the primary chloride permeation pathway in lysosomes. Nature. 2008;453(7196):788–92. 10.1038/nature06907 18449189

[110] Coffey EE, Beckel JM, Laties AM, Mitchell CH. Lysosomal alkalization and dysfunction in human fibroblasts with the Alzheimer's disease-linked presenilin 1 A246E mutation can be reversed with cAMP. Neuroscience. 2014;263:111–24. 10.1016/j.neuroscience.2014.01.001 24418614 PMC4028113

[111] Guha S, Liu J, Baltazar G, Laties AM, Mitchell CH. Rescue of compromised lysosomes enhances degradation of photoreceptor outer segments and reduces lipofuscin-like autofluorescence in retinal pigmented epithelial cells. Adv Exp Med Biol. 2014;801:105–11. 10.1007/978-1-4614-3209-8_14 24664687 PMC4163923

[112] Liu J, Lu W, Guha S, Baltazar GC, Coffey EE, Laties AM, et al Cystic fibrosis transmembrane conductance regulator contributes to reacidification of alkalinized lysosomes in RPE cells. Am J Physiol Cell Physiol. 2012;303(2):C160–9. 10.1152/ajpcell.00278.2011 22572847 PMC3404519

[113] DiCiccio JE, Steinberg BE. Lysosomal pH and analysis of the counter ion pathways that support acidification. J Gen Physiol. 2011;137(4):385–90. 10.1085/jgp.201110596 21402887 PMC3068279

[114] Noel S, Faveau C, Norez C, Rogier C, Mettey Y, Becq F. Discovery of pyrrolo[2,3-b]pyrazines derivatives as submicromolar affinity activators of wild type, G551D, and F508del cystic fibrosis transmembrane conductance regulator chloride channels. J Pharmacol Exp Ther. 2006;319(1):349–59. 10.1124/jpet.106.104521 16829626

[115] Ma T, Thiagarajah JR, Yang H, Sonawane ND, Folli C, Galietta LJ, et al Thiazolidinone CFTR inhibitor identified by high-throughput screening blocks cholera toxin-induced intestinal fluid secretion. J Clin Invest. 2002;110(11):1651–8. 10.1172/JCI16112 12464670 PMC151633

[116] Orfi L, Larive CK, LeVine SM. Physicochemical characterization of psychosine by 1H nuclear magnetic resonance and electron microscopy. Lipids. 1997;32(10):1035–40. 9358428 10.1007/s11745-997-0133-x

[117] Vanier MT. Lipid changes in Niemann-Pick disease type C brain: personal experience and review of the literature. Neurochem Res. 1999;24(4):481–9. 10227680 10.1023/a:1022575511354

[118] Hulkova H, Ledvinova J, Asfaw B, Koubek K, Kopriva K, Elleder M. Lactosylceramide in lysosomal storage disorders: a comparative immunohistochemical and biochemical study. Virchows Arch. 2005;447(1):31–44. 10.1007/s00428-005-1246-y 15918012

[119] Schueler UH, Kolter T, Kaneski CR, Blusztajn JK, Herkenham M, Sandhoff K, et al Toxicity of glucosylsphingosine (glucopsychosine) to cultured neuronal cells: a model system for assessing neuronal damage in Gaucher disease type 2 and 3. Neurobiol Dis. 2003;14(3):595–601. 14678774 10.1016/j.nbd.2003.08.016

[120] Sillence DJ, Puri V, Marks DL, Butters TD, Dwek RA, Pagano RE, et al Glucosylceramide modulates membrane traffic along the endocytic pathway. J Lipid Res. 2002;43(11):1837–45. 12401882 10.1194/jlr.m200232-jlr200

[121] Sillence DJ. Glucosylceramide modulates endolysosomal pH in Gaucher disease. Mol Genet Metab. 2013;109(2):194–200. 10.1016/j.ymgme.2013.03.015 23628459

[122] Farfel-Becker T, Vitner EB, Kelly SL, Bame JR, Duan J, Shinder V, et al Neuronal accumulation of glucosylceramide in a mouse model of neuronopathic Gaucher disease leads to neurodegeneration. Hum Mol Genet. 2014;23(4):843–54. 10.1093/hmg/ddt468 24064337 PMC3900102

[123] Dyatlovitskaya EV. The role of lysosphingolipids in the regulation of biological processes. Biochemistry (Mosc). 2007;72(5):479–84. 17573701 10.1134/s0006297907050033

[124] Nilsson O, Grabowski GA, Ludman MD, Desnick RJ, Svennerholm L. Glycosphingolipid studies of visceral tissues and brain from type 1 Gaucher disease variants. Clin Genet. 1985;27(5):443–50. 3924448 10.1111/j.1399-0004.1985.tb00229.x

[125] Igisu H, Suzuki K. Analysis of galactosylsphingosine (psychosine) in the brain. J Lipid Res. 1984;25(9):1000–6. 6491533

[126] Vanier M, Svennerholm L. Chemical pathology of Krabbe disease: the occurrence of psychosine and other neutral sphingoglycolipids. Adv Exp Med Biol. 1976;68:115–26. 937104 10.1007/978-1-4684-7735-1_8

[127] Vanier MT, Svennerholm L. Chemical pathology of Krabbe's disease. III. Ceramide-hexosides and gangliosides of brain. Acta Paediatr Scand. 1975;64(4):641–8. 1155084 10.1111/j.1651-2227.1975.tb03896.x

[128] Wang J, O'Bara MA, Pol SU, Sim FJ. CD133/CD140a-based isolation of distinct human multipotent neural progenitor cells and oligodendrocyte progenitor cells. Stem Cells Dev. 2013;22(15):2121–31. 10.1089/scd.2013.0003 23488628 PMC3715795

[129] Suzuki K, Yoshiyuki S. Globoid Cell Leucodystrophy (Krabbe's Disease): Deficiency of Galactocerebroside β-Galactosidase. *Proceedings of the National Academy of Sciences of the United States of America*† 1970 p. 302–9. 10.1073/pnas.66.2.302 PMC283044 5271165

[130] Li Y, Sands MS. Experimental therapies in the murine model of globoid cell leukodystrophy. Pediatr Neurol. 2014;51(5):600–6. 10.1016/j.pediatrneurol.2014.08.003 25240259 PMC4252788

[131] Itoh M, Hayashi M, Fujioka Y, Nagashima K, Morimatsu Y, Matsuyama H. Immunohistological study of globoid cell leukodystrophy. Brain Dev. 2002;24(5):284–90. 12142065 10.1016/s0387-7604(02)00057-8

[132] WHO Pharmaceutical Newsletter No. 9 & 12. Geneva, Switzerland: World Health Organization; 1999.

[133] Morinobu S, Fujimaki K, Okuyama N, Takahashi M, Duman RS. Stimulation of adenylyl cyclase and induction of brain-derived neurotrophic factor and TrkB mRNA by NKH477, a novel and potent forskolin derivative. J Neurochem. 1999;72(5):2198–205. 10217303 10.1046/j.1471-4159.1999.0722198.x

[134] Castelvetri LC, Givogri MI, Zhu H, Smith B, Lopez-Rosas A, Qiu X, et al Axonopathy is a compounding factor in the pathogenesis of Krabbe disease. Acta Neuropathol. 2011;122(1):35–48. 10.1007/s00401-011-0814-2 21373782 PMC3690521

[135] Walkley SU. Pathogenic mechanisms in lysosomal disease: a reappraisal of the role of the lysosome. Acta Paediatr. 2007;96(455):26–32. 10.1111/j.1651-2227.2007.00202.x 17391436

[136] Segatori L. Impairment of homeostasis in lysosomal storage disorders. IUBMB Life. 2014;66(7):472–7. 10.1002/iub.1288 25044960

[137] Noble M, Folts C, Scott N. Treating lysosomal storage disease. Google Patents; 2014.

[138] Villamil Giraldo AM, Appelqvist H, Ederth T, Öllinger K. Lysosomotropic agents: impact on lysosomal membrane permeabilization and cell death. Biochem Soc Trans. 2014;42(5):1460–4. 10.1042/BST20140145 25233432

[139] Kagedal K, Zhao M, Svensson I, Brunk UT. Sphingosine-induced apoptosis is dependent on lysosomal proteases. Biochem J. 2001;359(Pt 2):335–43. 11583579 10.1042/0264-6021:3590335 PMC1222151

[140] Shen D, Wang X, Li X, Zhang X, Yao Z, Dibble S, et al Lipid storage disorders block lysosomal trafficking by inhibiting a TRP channel and lysosomal calcium release. Nat Commun. 2012;3:731 10.1038/ncomms1735 22415822 PMC3347486

[141] Deriy LV, Gomez EA, Zhang G, Beacham DW, Hopson JA, Gallan AJ, et al Disease-causing mutations in the cystic fibrosis transmembrane conductance regulator determine the functional responses of alveolar macrophages. J Biol Chem. 2009;284(51):35926–38. 10.1074/jbc.M109.057372 19837664 PMC2791021

[142] Di A, Brown ME, Deriy LV, Li C, Szeto FL, Chen Y, et al CFTR regulates phagosome acidification in macrophages and alters bactericidal activity. Nat Cell Biol. 2006;8(9):933–44. 10.1038/ncb1456 16921366

[143] Steinberg BE, Huynh KK, Brodovitch A, Jabs S, Stauber T, Jentsch TJ, et al A cation counterflux supports lysosomal acidification. J Cell Biol. 2010;189(7):1171–86. 10.1083/jcb.200911083 20566682 PMC2894458

[144] Haggie PM, Verkman AS. Unimpaired lysosomal acidification in respiratory epithelial cells in cystic fibrosis. J Biol Chem. 2009;284(12):7681–6. 10.1074/jbc.M809161200 19136560 PMC2658062

[145] Barriere H, Bagdany M, Bossard F, Okiyoneda T, Wojewodka G, Gruenert D, et al Revisiting the role of cystic fibrosis transmembrane conductance regulator and counterion permeability in the pH regulation of endocytic organelles. Mol Biol Cell. 2009;20(13):3125–41. 10.1091/mbc.E09-01-0061 19420138 PMC2704163

[146] Weinert S, Jabs S, Supanchart C, Schweizer M, Gimber N, Richter M, et al Lysosomal pathology and osteopetrosis upon loss of H+-driven lysosomal Cl- accumulation. Science. 2010;328(5984):1401–3. 10.1126/science.1188072 20430974

[147] Teixeira CA, Miranda CO, Sousa VF, Santos TE, Malheiro AR, Solomon M, et al Early axonal loss accompanied by impaired endocytosis, abnormal axonal transport, and decreased microtubule stability occur in the model of Krabbe's disease. Neurobiol Dis. 2014;66:92–103. 10.1016/j.nbd.2014.02.012 24607884 PMC4307018

[148] Bashir A, Haq E. Effect of psychosine on inducible nitric-oxide synthase expression under different culture conditions: implications for Krabbe disease. Eur Rev Med Pharmacol Sci. 2011;15(11):1282–7. 22195360

[149] Yu RK, Ariga T, Yoshino H, Katoh-Semba R, Ren S. Differential Effects of Glycosphingolipids n Protein Kinase C Activity in PC12D Pheochromocytoma Cells. J Biomed Sci. 1994;1(4):229–36. 11725031 10.1007/BF02253307

[150] Graziano AC, Parenti R, Avola R, Cardile V. Krabbe disease: involvement of connexin43 in the apoptotic effects of sphingolipid psychosine on mouse oligodendrocyte precursors. Apoptosis. 2016;21(1):25–35. 10.1007/s10495-015-1183-4 26459425

[151] O'Sullivan C, Dev KK. Galactosylsphingosine (psychosine)-induced demyelination is attenuated by sphingosine 1-phosphate signalling. J Cell Sci. 2015;128(21):3878–87. 10.1242/jcs.169342 26359302

[152] Hawkins-Salsbury JA, Qin EY, Reddy AS, Vogler CA, Sands MS. Oxidative stress as a therapeutic target in globoid cell leukodystrophy. Exp Neurol. 2012;237(2):444–52. 10.1016/j.expneurol.2012.07.013 22849820 PMC3443330

[153] Tohyama J, Matsuda J, Suzuki K. Psychosine is as potent an inducer of cell death as C6-ceramide in cultured fibroblasts and in MOCH-1 cells. Neurochem Res. 2001;26(6):667–71. 11519726 10.1023/a:1010991420942

[154] Hans M, Pusch A, Dai L, Racké K, Swandulla D, Gieselmann V, et al Lysosulfatide regulates the motility of a neural precursor cell line via calcium-mediated process collapse. Neurochem Res. 2009;34(3):508–17. 10.1007/s11064-008-9813-7 18719997

[155] Kanazawa T, Kozutsumi Y. [Cytokinesis inhibition by glycosphingolipid, psychosine]. Tanpakushitsu Kakusan Koso. 2003;48(8 Suppl):1158–63. 12807024

[156] Nofer JR, Fobker M, H√∂bbel G, Voss R, Wolinska I, Tepel M, et al Activation of phosphatidylinositol-specific phospholipase C by HDL-associated lysosphingolipid. Involvement in mitogenesis but not in cholesterol efflux. Biochemistry. 2000;39(49):15199–207. 11106499 10.1021/bi001162a

[157] Sueyoshi N, Maehara T, Ito M. Apoptosis of Neuro2a cells induced by lysosphingolipids with naturally occurring stereochemical configurations. J Lipid Res. 2001;42(8):1197–202. 11483620

[158] Sakano S, Takemura H, Yamada K, Imoto K, Kaneko M, Ohshika H. Ca2+ mobilizing action of sphingosine in Jurkat human leukemia T cells. Evidence that sphingosine releases Ca2+ from inositol trisphosphate- and phosphatidic acid-sensitive intracellular stores through a mechanism independent of inositol trisphosphate. J Biol Chem. 1996;271(19):11148–55. 8626660 10.1074/jbc.271.19.11148

[159] Strasberg P. Cerebrosides and psychosine disrupt mitochondrial functions. Biochem Cell Biol. 1986;64(5):485–9. 3718715 10.1139/o86-067

[160] Van Veldhoven PP, Mannaerts GP, Declercq P, Baes M. Do sphingoid bases interact with the peroxisome proliferator activated receptor alpha (PPAR-alpha)? Cell Signal. 2000;12(7):475–9. 10989283 10.1016/s0898-6568(00)00092-9

[161] Tapasi S, Padma P, Setty OH. Effect of psychosine on mitochondrial function. Indian J Biochem Biophys. 1998;35(3):161–5. 9803665

[162] Liu R, Farach-Carson MC, Karin NJ. Effects of sphingosine derivatives on MC3T3-E1 pre-osteoblasts: psychosine elicits release of calcium from intracellualr stores. Biochem Biophys Res Commun. 1995;214(2):676–84. 7677781 10.1006/bbrc.1995.2339

[163] Sugiyama E, Uemura K, Hara A, Taketomi T. Effects of various lysosphingolipids on cell growth, morphology and lipid composition in three neuroblastoma cell lines. Biochem Biophys Res Commun. 1990;169(2):673–9. 2357225 10.1016/0006-291x(90)90383-x

[164] Fiore S, Nicolaou KC, Caulfield T, Kataoka H, Serhan CN. Evaluation of synthetic sphingosine, lysosphingolipids and glycosphingolipids as inhibitors of functional responses of human neutrophils. Biochem J. 1990;266(1):25–31. 2155608 10.1042/bj2660025 PMC1131091

[165] Hakomori S, Igarashi Y. Gangliosides and glycosphingolipids as modulators of cell growth, adhesion, and transmembrane signaling. Adv Lipid Res. 1993;25:147–62. 8396311

[166] Angka L, Lee EA, Rota SG, Hanlon T, Sukhai M, Minden M, et al Glucopsychosine increases cytosolic calcium to induce calpain-mediated apoptosis of acute myeloid leukemia cells. Cancer Lett. 2014;348(1–2):29–37. 10.1016/j.canlet.2014.03.003 24631520

[167] Lloyd-Evans E, Pelled D, Riebeling C, Bodennec J, de-Morgan A, Waller H, et al Glucosylceramide and glucosylsphingosine modulate calcium mobilization from brain microsomes via different mechanisms. J Biol Chem. 2003;278(26):23594–9. 10.1074/jbc.M300212200 12709427

[168] Im DS, Heise CE, Nguyen T, O'Dowd BF, Lynch KR. Identification of a molecular target of psychosine and its role in globoid cell formation. J Cell Biol. 2001;153(2):429–34. 11309421 10.1083/jcb.153.2.429 PMC2169470

[169] Pasqui AL, Di Renzo M, Auteri A, Federico G, Puccetti L. Increased TNF-alpha production by peripheral blood mononuclear cells in patients with Krabbe's disease: effect of psychosine. Eur J Clin Invest. 2007;37(9):742–5. 10.1111/j.1365-2362.2007.01850.x 17696965

[170] Katoh N. Inhibition by sulfatide of 21-kDa protein phosphorylation by protein kinase C in cow mammary gland and its reversal by phosphatidylserine. J Vet Med Sci. 2004;66(7):821–5. 15297754 10.1292/jvms.66.821

[171] Belleri M, Ronca R, Coltrini D, Nico B, Ribatti D, Poliani PL, et al Inhibition of angiogenesis by β-galactosylceramidase deficiency in globoid cell leukodystrophy. Brain. 2013;136(Pt 9):2859–75. 10.1093/brain/awt215 23983033 PMC3754455

[172] Igisu H, Hamasaki N, Ito A, Ou W. Inhibition of cytochrome c oxidase and hemolysis caused by lysosphingolipids. Lipids. 1988;23(4):345–8. 2840545 10.1007/BF02537346

[173] Kanazawa T, Nakamura S, Momoi M, Yamaji T, Takematsu H, Yano H, et al Inhibition of cytokinesis by a lipid metabolite, psychosine. J Cell Biol. 2000;149(4):943–50. 10811833 10.1083/jcb.149.4.943 PMC2174564

[174] Lloyd-Evans E, Pelled D, Riebeling C, Futerman AH. Lyso-glycosphingolipids mobilize calcium from brain microsomes via multiple mechanisms. Biochem J. 2003;375(Pt 3):561–5. 10.1042/BJ20030613 12917012 PMC1223730

[175] Meyer zu Heringdorf D, Jakobs KH. Lysophospholipid receptors: signalling, pharmacology and regulation by lysophospholipid metabolism. Biochim Biophys Acta. 2007;1768(4):923–40. 10.1016/j.bbamem.2006.09.026 17078925

[176] Gr√§ler MH, Goetzl EJ. Lysophospholipids and their G protein-coupled receptors in inflammation and immunity. Biochim Biophys Acta. 2002;1582(1–3):168–74. 12069825 10.1016/s1388-1981(02)00152-x

[177] Smith BR, Santos MB, Marshall MS, Cantuti-Castelvetri L, Lopez-Rosas A, Li G, et al Neuronal inclusions of α-synuclein contribute to the pathogenesis of Krabbe disease. J Pathol. 2014;232(5):509–21. 10.1002/path.4328 24415155 PMC3977150

[178] Vartanian T, Dawson G, Soliven B, Nelson DJ, Szuchet S. Phosphorylation of myelin basic protein in intact oligodendrocytes: inhibition by galactosylsphingosine and cyclic AMP. Glia. 1989;2(5):370–9. 10.1002/glia.440020509 2478466

[179] Tomura H, Mogi C, Sato K, Okajima F. Proton-sensing and lysolipid-sensitive G-protein-coupled receptors: a novel type of multi-functional receptors. Cell Signal. 2005;17(12):1466–76. 10.1016/j.cellsig.2005.06.002 16014326

[180] Ahn SH, Lee SY, Baek JE, Park SY, Lee YS, Kim H, et al Psychosine inhibits osteoclastogenesis and bone resorption via G protein-coupled receptor 65. J Endocrinol Invest. 2015;38(8):891–9. 10.1007/s40618-015-0276-9 25841894

[181] Contreras MA, Haq E, Uto T, Singh I, Singh AK. Psychosine-induced alterations in peroxisomes of twitcher mouse liver. Arch Biochem Biophys. 2008;477(2):211–8. 10.1016/j.abb.2008.06.012 18602885 PMC2654593

[182] Formichi P, Radi E, Battisti C, Pasqui A, Pompella G, Lazzerini PE, et al Psychosine-induced apoptosis and cytokine activation in immune peripheral cells of Krabbe patients. J Cell Physiol. 2007;212(3):737–43. 10.1002/jcp.21070 17458901

[183] Mitchison TJ. Psychosine, cytokinesis, and orphan receptors. Unexpected connections. J Cell Biol. 2001;153(2):F1–3. 11309405 10.1083/jcb.153.2.f1 PMC2169451

[184] Won JS, Kim J, Paintlia MK, Singh I, Singh AK. Role of endogenous psychosine accumulation in oligodendrocyte differentiation and survival: implication for Krabbe disease. Brain Res. 2013;1508:44–52. 10.1016/j.brainres.2013.02.024 23438514 PMC3996506

[185] Miguel BG, Calcerrada MC, Catalån RE, MartÍnez AM. Sphingolipid derivatives modulate intracellular Ca2+ in rat synaptosomes. Acta Neurobiol Exp (Wars). 2001;61(2):113–7. 11512408 10.55782/ane-2001-1391

[186] Sender Baum MG, Ahrén KE. Sphingosine and psychosine, suggested inhibitors of protein kinase C, inhibit LH effects in rat luteal cells. Mol Cell Endocrinol. 1988;60(2–3):127–35. 2463943 10.1016/0303-7207(88)90171-2

[187] Wang JQ, Kon J, Mogi C, Tobo M, Damirin A, Sato K, et al TDAG8 is a proton-sensing and psychosine-sensitive G-protein-coupled receptor. J Biol Chem. 2004;279(44):45626–33. 10.1074/jbc.M406966200 15326175

[188] Guha S, Coffey EE, Lu W, Lim JC, Beckel JM, Laties AM, et al Approaches for detecting lysosomal alkalinization and impaired degradation in fresh and cultured RPE cells: evidence for a role in retinal degenerations. Exp Eye Res. 2014;126:68–76. 10.1016/j.exer.2014.05.013 25152362 PMC4143779

[189] Bourdenx M, Daniel J, Genin E, Soria FN, Blanchard-Desce M, Bezard E, et al Nanoparticles restore lysosomal acidification defects: Implication for Parkinson and other lysosomal-related diseases. Autophagy. 2016:0. 10.1080/15548627.2015.1136769 PMC4835967 26761717

[190] Guha S, Baltazar GC, Tu LA, Liu J, Lim JC, Lu W, et al Stimulation of the D5 dopamine receptor acidifies the lysosomal pH of retinal pigmented epithelial cells and decreases accumulation of autofluorescent photoreceptor debris. J Neurochem. 2012;122(4):823–33. 10.1111/j.1471-4159.2012.07804.x 22639870 PMC3408960

[191] Wang MX, Cheng XY, Jin M, Cao YL, Yang YP, Wang JD, et al TNF compromises lysosome acidification and reduces α-synuclein degradation via autophagy in dopaminergic cells. Exp Neurol. 2015;271:112–21. 10.1016/j.expneurol.2015.05.008 26001614

[192] Chwieralski CE, Welte T, Bühling F. Cathepsin-regulated apoptosis. Apoptosis. 2006;11(2):143–9. 10.1007/s10495-006-3486-y 16502253

[193] Orrenius S, Zhivotovsky B, Nicotera P. Regulation of cell death: the calcium-apoptosis link. Nat Rev Mol Cell Biol. 2003;4(7):552–65. 10.1038/nrm1150 12838338

[194] Stoka V, Turk V, Turk B. Lysosomal cysteine cathepsins: signaling pathways in apoptosis. Biol Chem. 2007;388(6):555–60. 10.1515/BC.2007.064 17552902

[195] Taniike M, Suzuki K. Proliferative capacity of oligodendrocytes in the demyelinating twitcher spinal cord. J Neurosci Res. 1995;40(3):325–32. 10.1002/jnr.490400306 7745626

[196] Orsini JJ, Kay DM, Saavedra-Matiz CA, Wenger DA, Duffner PK, Erbe RW, et al Newborn screening for Krabbe disease in New York State: the first eight years' experience. Genet Med. 2016. 10.1038/gim.2015.211 26795590

[197] Dimmock DP. Should states adopt newborn screening for early infantile Krabbe disease? Genet Med. 2016. 10.1038/gim.2016.6 26845105

[198] Holopainen JM, Saarikoski J, Kinnunen PK, Järvelä I. Elevated lysosomal pH in neuronal ceroid lipofuscinoses (NCLs). Eur J Biochem. 2001;268(22):5851–6. 11722572 10.1046/j.0014-2956.2001.02530.x

[199] Tofaris GK. Lysosome-dependent pathways as a unifying theme in Parkinson's disease. Mov Disord. 2012;27(11):1364–9. 10.1002/mds.25136 22927213

[200] Nixon RA. The role of autophagy in neurodegenerative disease. Nat Med. 2013;19(8):983–97. 10.1038/nm.3232 23921753

[201] De Strooper B. Proteases and proteolysis in Alzheimer disease: a multifactorial view on the disease process. Physiol Rev. 2010;90(2):465–94. 10.1152/physrev.00023.2009 20393191

[202] De Kimpe L, van Haastert ES, Kaminari A, Zwart R, Rutjes H, Hoozemans JJ, et al Intracellular accumulation of aggregated pyroglutamate amyloid beta: convergence of aging and Aβ pathology at the lysosome. Age (Dordr). 2013;35(3):673–87. 22477259 10.1007/s11357-012-9403-0 PMC3636379

[203] Ditaranto K, Tekirian TL, Yang AJ. Lysosomal membrane damage in soluble Abeta-mediated cell death in Alzheimer's disease. Neurobiol Dis. 2001;8(1):19–31. 10.1006/nbdi.2000.0364 11162237

[204] Hui L, Chen X, Geiger JD. Endolysosome involvement in LDL cholesterol-induced Alzheimer's disease-like pathology in primary cultured neurons. Life Sci. 2012;91(23–24):1159–68. 10.1016/j.lfs.2012.04.039 22580286 PMC3431446

[205] Noonan J, Tanveer R, Klompas A, Gowran A, McKiernan J, Campbell VA. Endocannabinoids prevent β-amyloid-mediated lysosomal destabilization in cultured neurons. J Biol Chem. 2010;285(49):38543–54. 10.1074/jbc.M110.162040 20923768 PMC2992287

[206] Kanazirska MV, Fuchs PM, Chen L, Lal S, Verma J, Vassilev PM. Beneficial effects of lysosome-modulating and other pharmacological and nanocarrier agents on amyloid-beta-treated cells. Curr Pharm Biotechnol. 2012;13(15):2761–7. 23072393 10.2174/138920112804724909

[207] Bahr BA, Wisniewski ML, Butler D. Positive lysosomal modulation as a unique strategy to treat age-related protein accumulation diseases. Rejuvenation Res. 2012;15(2):189–97. 10.1089/rej.2011.1282 22533430 PMC3332372

[208] Butler D, Hwang J, Estick C, Nishiyama A, Kumar SS, Baveghems C, et al Protective effects of positive lysosomal modulation in Alzheimer's disease transgenic mouse models. PLoS ONE. 2011;6(6):e20501 10.1371/journal.pone.0020501 21695208 PMC3112200

[209] Yang DS, Stavrides P, Mohan PS, Kaushik S, Kumar A, Ohno M, et al Therapeutic effects of remediating autophagy failure in a mouse model of Alzheimer disease by enhancing lysosomal proteolysis. Autophagy. 2011;7(7):788–9. 10.4161/auto.7.7.15596 21464620 PMC3359468

[210] Avrahami L, Farfara D, Shaham-Kol M, Vassar R, Frenkel D, Eldar-Finkelman H. Inhibition of glycogen synthase kinase-3 ameliorates Œ≤-amyloid pathology and restores lysosomal acidification and mammalian target of rapamycin activity in the Alzheimer disease mouse model: in vivo and in vitro studies. J Biol Chem. 2013;288(2):1295–306. 10.1074/jbc.M112.409250 23155049 PMC3543013

[211] Balducci C, Pierguidi L, Persichetti E, Parnetti L, Sbaragli M, Tassi C, et al Lysosomal hydrolases in cerebrospinal fluid from subjects with Parkinson's disease. Mov Disord. 2007;22(10):1481–4. 10.1002/mds.21399 17546678

[212] Gegg ME, Burke D, Heales SJ, Cooper JM, Hardy J, Wood NW, et al Glucocerebrosidase deficiency in substantia nigra of parkinson disease brains. Ann Neurol. 2012;72(3):455–63. 10.1002/ana.23614 23034917 PMC3638323

[213] Perry SW, Norman JP, Litzburg A, Gelbard HA. Antioxidants are required during the early critical period, but not later, for neuronal survival. J Neurosci Res. 2004;78(4):485–92. 10.1002/jnr.20272 15389829

[214] Frost EE, Milner R, Ffrench-Constant C. Migration assays for oligodendrocyte precursor cells. Methods Mol Biol. 2000;139:265–78. 10.1385/1-59259-063-2:265 10840794

[215] Milner R, Edwards G, Streuli C, Ffrench-Constant C. A role in migration for the alpha V beta 1 integrin expressed on oligodendrocyte precursors. J Neurosci. 1996;16(22):7240–52. 8929432 10.1523/JNEUROSCI.16-22-07240.1996 PMC6578950

[216] Li Z, Dong T, Pröschel C, Noble M. Chemically diverse toxicants converge on Fyn and c-Cbl to disrupt precursor cell function. PLoS Biol. 2007;5(2):e35 10.1371/journal.pbio.0050035 17298174 PMC1790953

[217] Bielawski J, Pierce JS, Snider J, Rembiesa B, Szulc ZM, Bielawska A. Comprehensive quantitative analysis of bioactive sphingolipids by high-performance liquid chromatography-tandem mass spectrometry. Methods Mol Biol. 2009;579:443–67. 10.1007/978-1-60761-322-0_22 19763489

