# Supplementary figures and images for: Lysosomal Re-acidification Prevents Lysosphingolipid-Induced Lysosomal Impairment and Cellular Toxicity
Source: PLoS Biol. 2016 Dec 15;14(12):e1002583. doi: 10.1371/journal.pbio.1002583 (PMC5169359; doi:10.1371/journal.pbio.1002583)

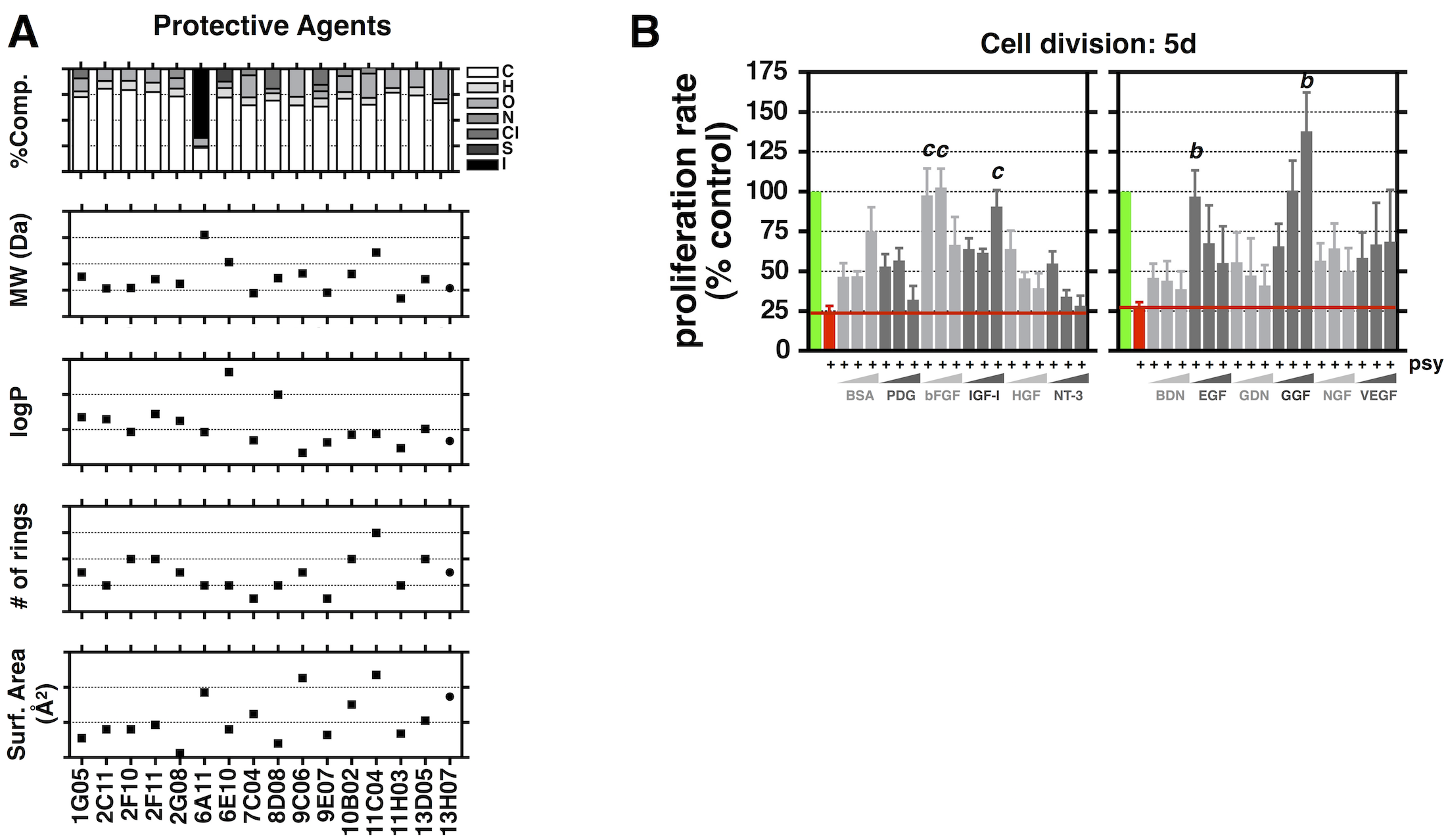

Supplement: S2 Fig — (A) Physicochemical characterization of small molecules that reduce Psy-induced (D) cell death or (E) suppression of division, including atomic composition (% by mass), molecular weight (Daltons), logP partition coefficient, number of ring structures, and surface area (Å2). (B) Quantification of cell division of rat O-2A/OPCs exposed to 1.5 μM Psy for 5 d, with and without the indicated growth factors at 10, 33, or 100 ng/mL. Data for all graphs displayed as mean ± SEM; ap < 0.05, bp < 0.01, cp < 0.001 versus Psy-only treatment. See S1 and S2 Tables for drugs and concentrations used. Data presented in this figure can be found in S1 Data. (TIFF) [file pbio.1002583.s003.tiff]

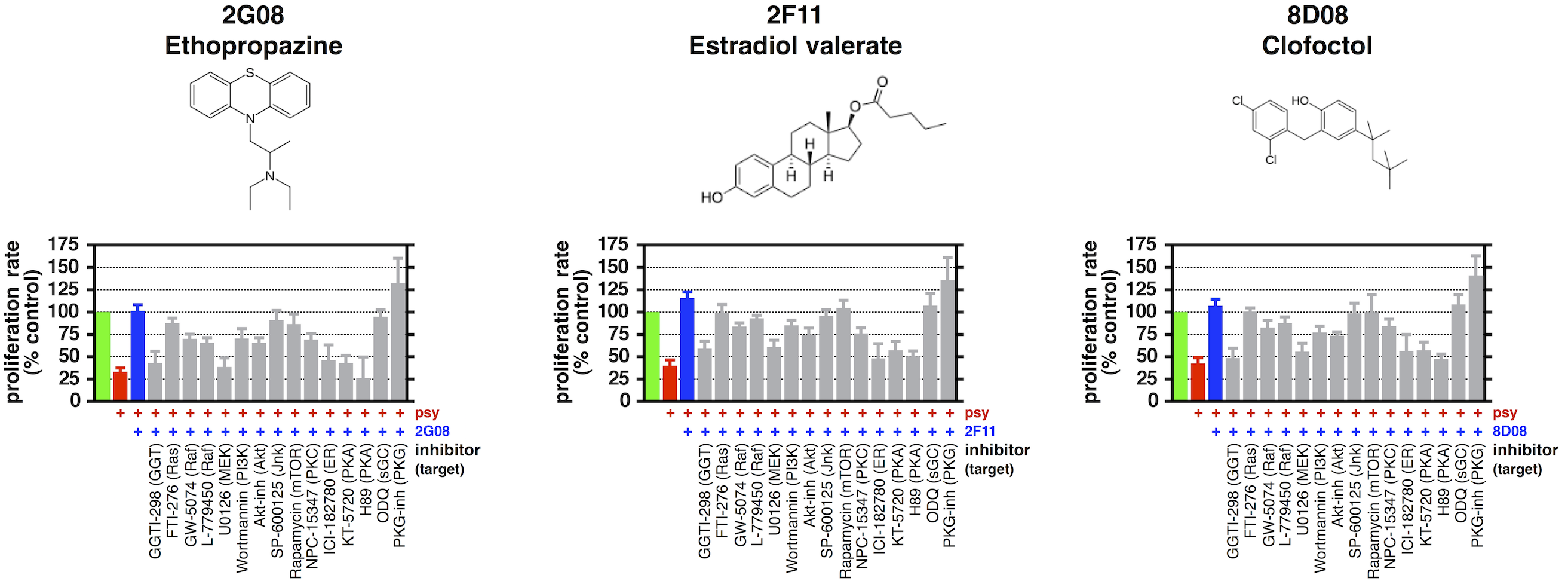

Supplement: S3 Fig — Representative “fingerprints of protection” for the functionally and structurally unrelated candidate drugs 2G08, 2F11, and 8D08. Data represent mean ± SEM. See also See S1 and S2 Tables for drugs and concentrations, and S3 Table for details on the “fingerprinting” screen. Data presented in this figure can be found in S1 Data. (TIFF) [file pbio.1002583.s004.tiff]

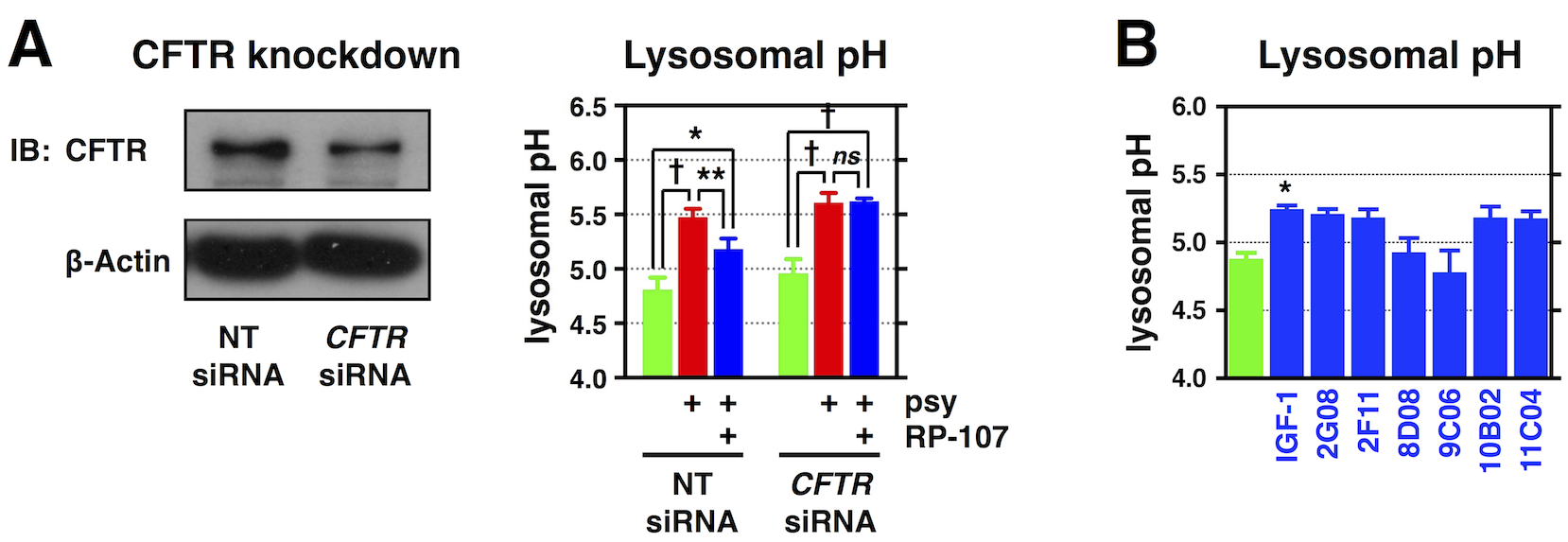

Supplement: S4 Fig — (A) A representative western blot of CFTR knockdown versus NT controls in rat O-2A/OPCs, 4 d post transfection. Quantification of lysosomal pH in rat O-2A/OPCs, with or without CFTR knockdown (5 d post transfection), exposed to 1 μM Psy or 1 μM Psy and 333 nM RP-107 for 24 h. (B) Quantification of lysosomal pH of rat O-2A/OPCs exposed to the indicated drugs for 24 h in the absence of Psy. Data for all graphs displayed as mean ± SEM; *p < 0.05, **p < 0.01, †p < 0.001. See S1 and S2 Tables for drugs and concentrations used. Data presented in this figure can be found in S1 Data. (TIFF) [file pbio.1002583.s005.tiff]

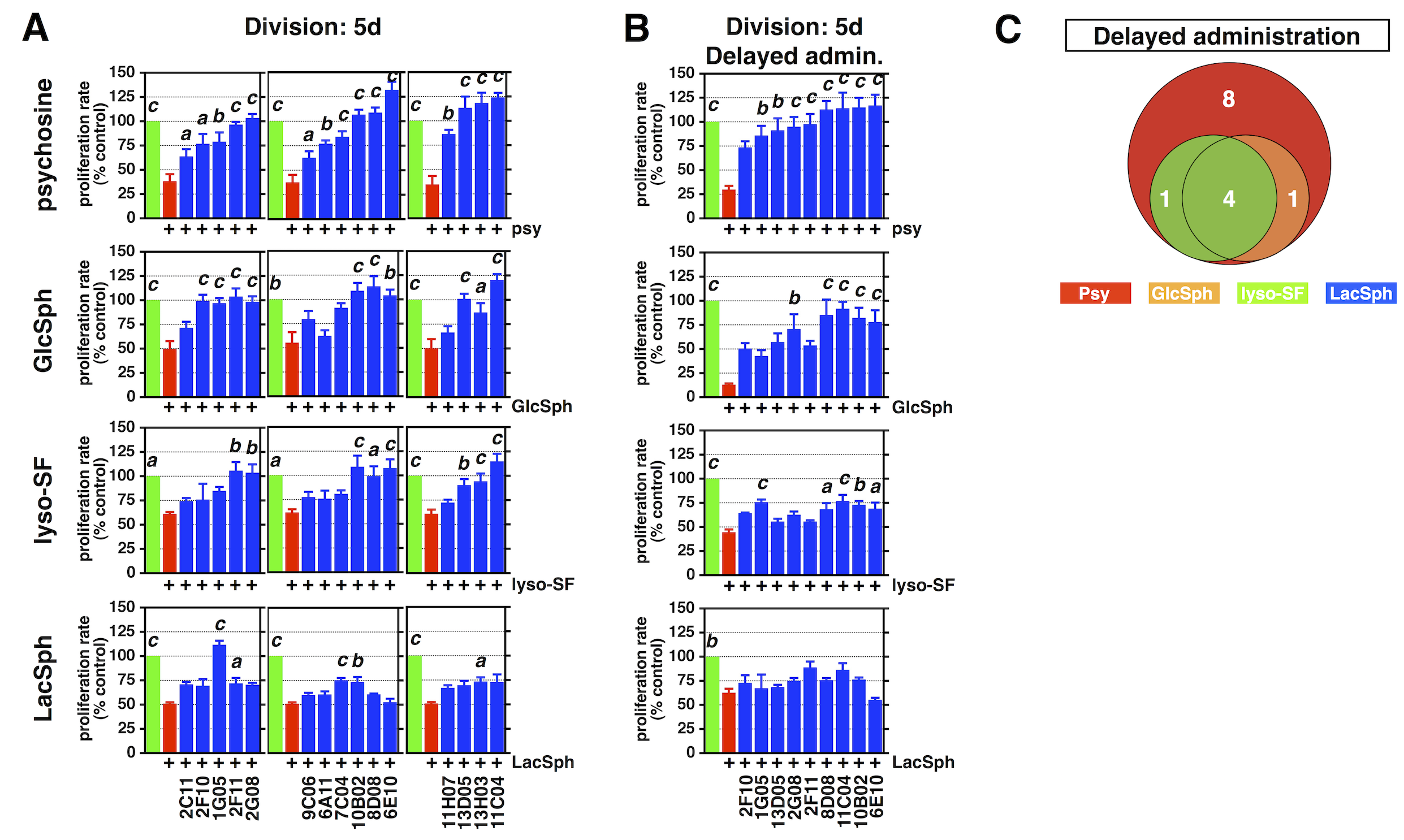

Supplement: S5 Fig — (A) Proliferation analysis of rat O-2A/OPCs exposed to 1.5 μM Psy, 1 μM GlcSph, 3 μM Lyso-SF, or 12 μM LacSph for 5 d, with and without the indicated protective agents. (B) Proliferation analysis of rat O-2A/OPCs exposed to 1.5 μM Psy, 1 μM GlcSph, 3 μM Lyso-SF, or 12 μM LacSph for 5 d, with and without the indicated protective agents, which were administered 2 d after the indicated lyso-lipid. (C) Venn diagram summarizing (B) for all lyso-lipids. Data for all graphs displayed as mean ± SEM; ap < 0.05, bp < 0.01, cp < 0.001 versus lipid-only treatment. See S1 and S2 Tables for drugs and concentrations used. Data presented in this figure can be found in S1 Data. (TIFF) [file pbio.1002583.s006.tiff]

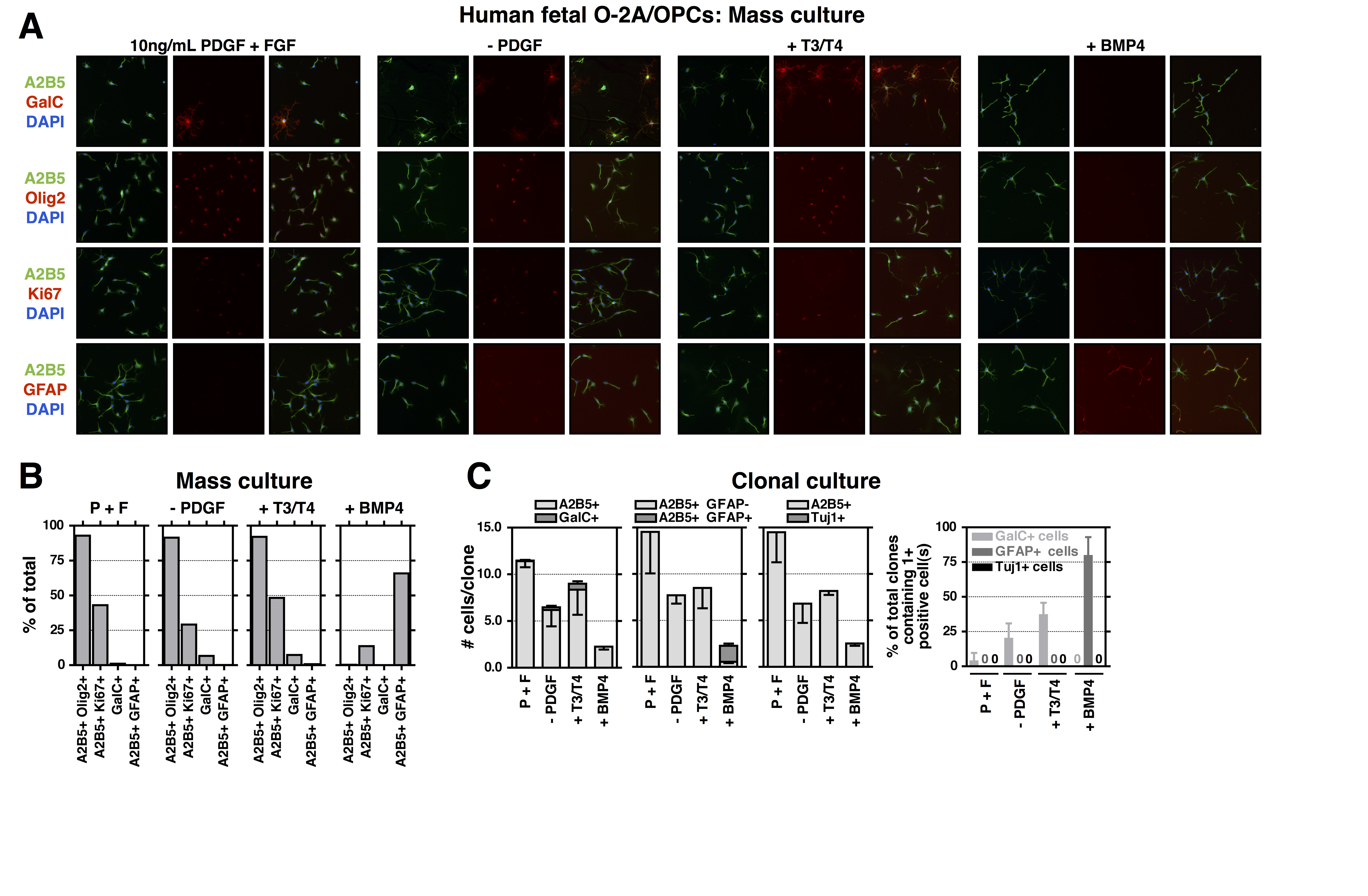

Supplement: S6 Fig — (A) Representative immunofluorescent images of human fetal O-2A/OPCs maintained in 10 ng/mL PDGF + 10 ng/mL bFGF (“PDGF+FGF”); 100 pg/mL PDGF (“- PDGF”); 1 ng/mL PDGF + 40 ng/mL T3/T4 (“+T3/T4”); and 1 ng/mL PDGF + 10 ng/mL BMP4 (“+BMP4”) for 5 d in mass culture. A2B5+: glial progenitor cells; GalC+: OLs; GFAP: astrocytes; A2B5+/GFAP+: Type-2 astrocytes; Ki67+: mitotically active cells; Olig2+: oligodendroglial-lineage cells. (B) Quantification of (A). (C) Quantification of human fetal O-2A/OPCs maintained as in (A) for 5 d, except at clonal density. The mean number of cells immunopositive for the indicated stain per clone, as well as the percentage of clones containing at least one immunopositive cell, are reported. Note that NeuN+ or Tuj1+ neurons were never detected in mass or clonal culture. Data for all graphs displayed as mean ± SD for one GW20 human sample. All experiments were repeated in four human GW19-21 samples with comparable results. Data presented in this figure can be found in S1 Data. (TIFF) [file pbio.1002583.s007.tiff]

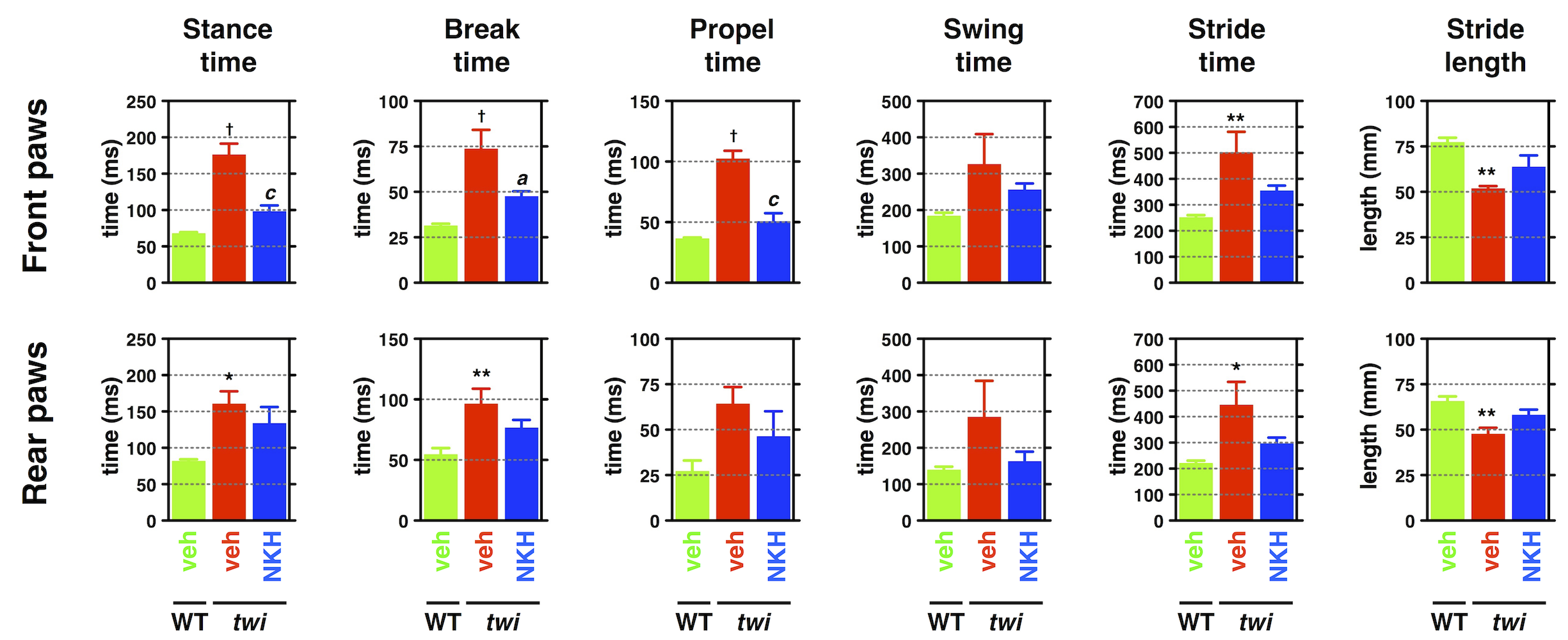

Supplement: S7 Fig — Quantification of gait for P25 vehicle-treated WT (n = 3–4), vehicle-treated twitcher mice (n = 3), and NKH-treated twitcher mice (n = 4), including measurements of stance, break, propel, swing, and stride time, as well stride length, for front and rear paws. Data for all graphs displayed as mean ± SEM; *p < 0.05, **p < 0.01, †p < 0.001 versus WT; ap < 0.05, cp < 0.001 versus vehicle-treated twitcher. Data presented in this figure can be found in S1 Data. (TIFF) [file pbio.1002583.s008.tiff]
