# Supplementary material for: Lysosomal Re-acidification Prevents Lysosphingolipid-Induced Lysosomal Impairment and Cellular Toxicity
Source: PLoS Biol. 2016 Dec 15;14(12):e1002583. doi: 10.1371/journal.pbio.1002583 (PMC5169359; doi:10.1371/journal.pbio.1002583)
Supplement: S1 Table — (DOCX) [file pbio.1002583.s012.docx]

**Chemical Structures of Lead Protective Compounds**

| **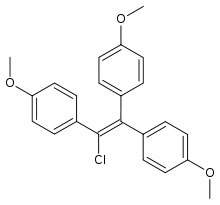** | | **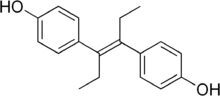** | | **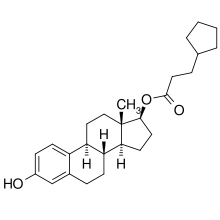** | | **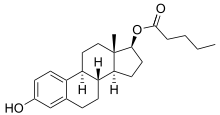** | |
| --- | --- | --- | --- | --- | --- | --- | --- |
| **1G05**  **Chlorotrianisene** | | **2C11**  **Diethylstilbestrol** | | **2F10**  **Estradiol cypionate** | | **2F11**  **Estradiol valerate** | |
| **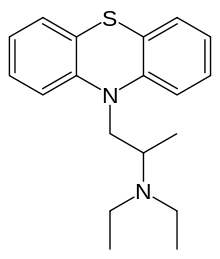** | | **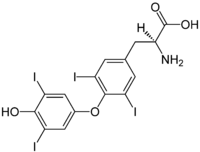** | | **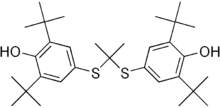** | | **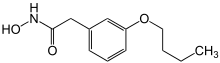** | |
| **2G08**  **Ethopropazine** | | **6A11**  **Thyroxine** | | **6E10**  **Probucol** | | **7C04**  **Bufexamac** | |
| **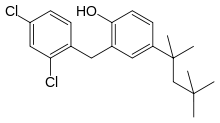** | | **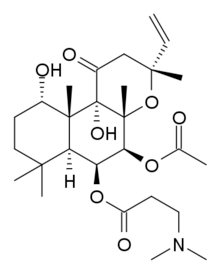** | | **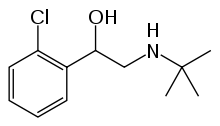** | | **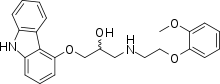** | |
| **8D08**  **Clofoctol** | | **9C06**  **Colforsin (NKH-477)** | | **9E07**  **Tulobuterol** | | **10B02**  **Carvedilol** | |
| **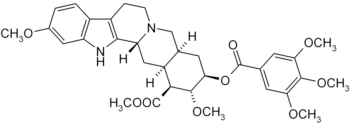** | | **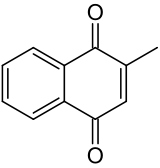** | | 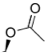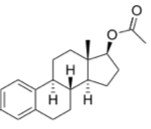 | | **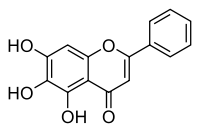** | |
| **11C04**  **Reserpine** | | **11H07**  **Menadione** | | **13D05**  **Estradiol diacetate** | | **13H03**  **Baicalein** | |
| **NON-**  **HITS** | **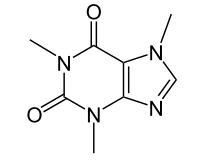** | | **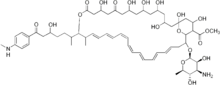** | | ***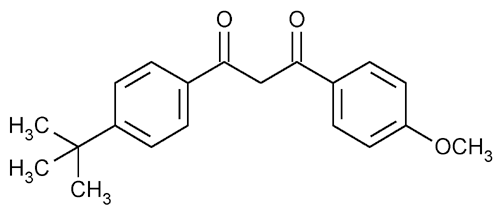*** | | 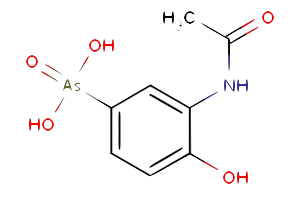 |
|  | ***1E04***  ***Caffeine*** | | ***9A06***  ***Mepartricin*** | | ***9H10***  ***Avobenzone*** | | ***5F05***  ***Acetarsol*** |
