# Supplementary material for: Lysosomal Re-acidification Prevents Lysosphingolipid-Induced Lysosomal Impairment and Cellular Toxicity
Source: PLoS Biol. 2016 Dec 15;14(12):e1002583. doi: 10.1371/journal.pbio.1002583 (PMC5169359; doi:10.1371/journal.pbio.1002583)
Supplement: S2 Table — (DOCX) [file pbio.1002583.s013.docx]

**Lead Protective Compounds**

| **Library No.** | **Compound name** | **Optimal concentration** | | **FDA** | **BBB** | **Use** |
| --- | --- | --- | --- | --- | --- | --- |
|  |  | **rOPCs** | **hOPCs** |  |  |  |
| **1G05** | Chlorotrianisene | 100nM | 1μM | ✓ | ✓ | nonsteroidal synthetic estrogen (HRT) |
| **2C11** | Diethylstilbestrol | 1μM | 1μM | ✓ | ✓ | nonsteroidal synthetic estrogen (HRT) |
| **2F10** | Estradiol cypionate | 1μM | 333nM | ✓ | ✓ | synthetic estrogen (HRT) |
| **2F11** | Estradiol valerate | 1μM | 1μM | ✓ | ✓ | synthetic estrogen (HRT) |
| **2G08** | Ethopropazine | 100nM | 333nM | ✓ | ✓ | anti-dyskinetic (Parkinson’s) |
| **6A11** | Thyroxine | 1μM | 3.3μM | ✓ | ✓ | anti-hyperlipidemic (thyroid hormone T4) |
| **6E10** | Probucol | 10μM | 333nM | ✓ | ✓ | anti-cholesterolemic |
| **7C04** | Bufexamac | 333nM | 10μM | (AU) | nd | anti-inflammatory (eczema) |
| **8D08** | Clofoctol | 100nM | 333nM | (EU) | nd | bacteriostatic antibiotic |
| **9C06** | Colforsin (NKH-477) | 100nM | 1μM | (JP) | ✓ | cAMP-elevating compound (forskolin) |
| **9E07** | Tulobuterol | 333nM | 10μM | (JP) | nd | adrenergic agonist (asthma, COPD) |
| **10B02** | Carvedilol | 3.3μM | 3.3μM | ✓ | ✓ | beta-blocker (congestive heart failure) |
| **11C04** | Reserpine | 3.3μM | 3.3μM | ✓ | ✓ | anti-hypertensive, anti-psychotic |
| **11H07** | Menadione | 333nM | 100nM | ✓ | ✓ | synthetic Vitamin K2 precursor |
| **13D05** | Estradiol diacetate | 1μM | 1μM | ✓ | ✓ | synthetic estrogen, contraceptive |
| **13H03** | Baicalein | 100nM | 333nM |  | ✓ | flavonoid, anti-inflammatory |
