# Supplementary material for: Lysosomal Re-acidification Prevents Lysosphingolipid-Induced Lysosomal Impairment and Cellular Toxicity
Source: PLoS Biol. 2016 Dec 15;14(12):e1002583. doi: 10.1371/journal.pbio.1002583 (PMC5169359; doi:10.1371/journal.pbio.1002583)
Supplement: S3 Table — (DOCX) [file pbio.1002583.s014.docx]

**Pharmacological inhibitor details from ‘fingerprinting’ screen**

| **Inhibitor** | **Concentration** | **Target** |
| --- | --- | --- |
| GGTI-298 | 333 nM | Geranylgeranyl transferase (Rac, Rho, etc) |
| FTI-276 | 33 nM | Farnesyl transferase (Ras) |
| L-779,450 | 333 nM | Raf-1 |
| GW-5074 | 333 nM | Raf-1 |
| U0126 | 1 μM | MEK |
| Wortmannin | 33 nM | PI3K |
| Akt1/2 inhibitor | 333 nM | Akt |
| SP-600125 | 333 nM | Jnk |
| Rapamycin | 100 fM | mTORC1 |
| NPC-15437 | 333 nM | Protein Kinase C |
| ICI-182790 | 3.3 μM | Estrogen receptor |
| KT-5720 | 100 nM | Protein Kinase A |
| H89 | 3.3 μM | Protein Kinase A |
| ODQ | 100 nM | Guanyl cyclase (sGC) |
| PKG inhibitor | 100 nM | Protein Kinase G |
