# Supplementary material for: Lysosomal Re-acidification Prevents Lysosphingolipid-Induced Lysosomal Impairment and Cellular Toxicity
Source: PLoS Biol. 2016 Dec 15;14(12):e1002583. doi: 10.1371/journal.pbio.1002583 (PMC5169359; doi:10.1371/journal.pbio.1002583)
Supplement: S4 Table — (DOCX) [file pbio.1002583.s015.docx]

**Concentrations of lipids used with rat and human OPCs**

|  | **LC50 concentration (5d)** | | **“Sublethal” concentration**  **(to 5d)** | |
| --- | --- | --- | --- | --- |
|  | **Rat OPC** | **Human OPC** | **Rat OPC** | **Human OPC** |
| **Psy** | 3.3μM | 2μM | 1μM | 1μM |
| **GlcSph** | 3μM | 2μM | 1μM | 1μM |
| **Lyso-SF** | 7μM | 2μM | 3μM | 1μM |
| **LacSph** | 16μM | 14μM | 12μM | 10μM |
